# Benchmarking AlphaFold for protein complex modeling reveals accuracy determinants

**DOI:** 10.1002/pro.4379

**Published:** 2022-07-13

**Authors:** Rui Yin, Brandon Y. Feng, Amitabh Varshney, Brian G. Pierce

**Affiliations:** ^1^ Institute for Bioscience and Biotechnology Research University of Maryland Rockville Maryland USA; ^2^ Department of Cell Biology and Molecular Genetics University of Maryland College Park Maryland USA; ^3^ Department of Computer Science University of Maryland College Park Maryland USA; ^4^ Marlene and Stewart Greenebaum Comprehensive Cancer Center University of Maryland School of Medicine Baltimore Maryland USA

## Abstract

High‐resolution experimental structural determination of protein–protein interactions has led to valuable mechanistic insights, yet due to the massive number of interactions and experimental limitations there is a need for computational methods that can accurately model their structures. Here we explore the use of the recently developed deep learning method, AlphaFold, to predict structures of protein complexes from sequence. With a benchmark of 152 diverse heterodimeric protein complexes, multiple implementations and parameters of AlphaFold were tested for accuracy. Remarkably, many cases (43%) had near‐native models (medium or high critical assessment of predicted interactions accuracy) generated as top‐ranked predictions by AlphaFold, greatly surpassing the performance of unbound protein–protein docking (9% success rate for near‐native top‐ranked models), however AlphaFold modeling of antibody–antigen complexes within our set was unsuccessful. We identified sequence and structural features associated with lack of AlphaFold success, and we also investigated the impact of multiple sequence alignment input. Benchmarking of a multimer‐optimized version of AlphaFold (AlphaFold‐Multimer) with a set of recently released antibody–antigen structures confirmed a low rate of success for antibody–antigen complexes (11% success), and we found that T cell receptor–antigen complexes are likewise not accurately modeled by that algorithm, showing that adaptive immune recognition poses a challenge for the current AlphaFold algorithm and model. Overall, our study demonstrates that end‐to‐end deep learning can accurately model many transient protein complexes, and highlights areas of improvement for future developments to reliably model any protein–protein interaction of interest.

## 1 INTRODUCTION

Protein–protein interactions are the basis of many critical and fundamental cellular and molecular processes, including inhibition or activation of enzymes, cellular signaling, and recognition of antigens by the adaptive immune system. High‐resolution structural characterization of these interactions provides insights into their molecular basis, as well as structure‐guided design of binding affinities and identification of inhibitors. However, structures for large numbers of molecular interactions remain undetermined experimentally, due to limitations in resources, and the challenges of structural determination techniques.

In response to this need, numerous predictive computational methods to model structures of protein–protein complexes have been developed over several decades, including protein docking methods that use unbound or modeled component structures as input to perform rigid‐body global searches in six dimensions,^1^, ^2^, ^3^, ^4^, ^5^ and template‐based modeling methods that generate models of complexes based on known structures.^6^, ^7^ Challenges for docking algorithms include side chain and backbone conformational changes between unbound and bound structures, large search spaces, and inability to capture key energetic features in grid‐based and other rapidly computable functions, leading to false positive models among top‐ranked models or lack of any near‐native models within large sets of predicted models. Developments such as explicit side chain flexibility during docking searches,^8^ use of normal mode analysis to represent protein flexibility,^9^, ^10^ clustering^11^, ^12^ or re‐scoring^13^, ^14^, ^15^, ^16^ docking models to improve ranking of near‐native models, and use of experimental data as restraints for docking^17^ have led to some improvement in docking success, and examples of these and other advances specifically designed to address the challenge posed by protein backbone flexibility are highlighted in a recent review.^18^ However, the Critical Assessment of Predicted Interactions (CAPRI) blind docking prediction experiment^19^ and several protein docking benchmarks,^20^, ^21^ which have enabled the systematic assessment of predictive docking performance, revealed persistent shortcomings of current computational docking approaches. Several protein–protein complex targets had no accurate model generated by any teams in a set of recent CAPRI rounds,^22^ while benchmarking of multiple docking algorithms in 2015 showed no accurate models within sets of top‐ranked predictions for many of the test cases.^20^ A more recent benchmarking study with 67 antibody–antigen docking test cases highlighted the limited success for current global docking approaches, which was more pronounced for cases with more conformational changes between unbound and bound structures.^23^

The recently developed AlphaFold algorithm (AlphaFold v.2.0) performs end‐to‐end modeling with a deep neural network to generate structural models from sequence,^24^ showing unprecedentedly high modeling accuracy and substantially surpassing the performance of other teams in the most recent critical assessment of structural prediction (CASP) round (CASP14).^25^ An important element of the AlphaFold algorithm is the combinatorial use of row‐wise, column‐wise and triangle self‐attention to iteratively infer residue distance and evolutionary information from multiple sequence alignments (MSAs), building on previous work demonstrating the use of coevolution in contact prediction.^26^, ^27^ The resulting feature representations are further processed by a geometry‐aware attention‐based structure module that rotates and translates each residue to produce a 3D protein structure prediction. After the remarkable success of AlphaFold in CASP14, a separate team of researchers developed RoseTTAFold,^28^ which likewise takes MSAs as input, and outputs 3D structural predictions, using attention‐based deep learning architecture. Unlike AlphaFold, RoseTTAFold utilizes a “three‐track” approach, allowing for concurrent updates within and in‐between 1D amino acid sequence, 2D pairwise distances and orientations between residues, and 3D structural coordinates.

The reported capability to model homomultimers,^24^ as well as a recently reported adaptation of AlphaFold to enable modeling of heteroprotein assemblies,^29^ raises the question of how accurately AlphaFold can model transient heteroprotein complexes, including classes of complexes that have challenged previously developed and currently available docking approaches. As the AlphaFold deep learning model was trained using experimentally determined structures of individual protein chains,^24^ and its accuracy was partly enabled by residue distances within tertiary structures inferred from MSA, it is not clear whether it can reliably generate protein–protein interface structures, particularly for transient protein complexes which have distinct physicochemical properties than protein interiors^30^ and obligate protein–protein interfaces,^31^, ^32^ as well as a lack of explicit MSA signal from pairs of residues across the protein–protein interface in the sequences.

Here we report a systematic assessment of the accuracy of AlphaFold in performing end‐to‐end modeling of transient protein complexes, using 152 heterodimeric test cases from Protein–Protein Docking Benchmark version 5.5 (BM5.5)^20^, ^23^ which represent three previously established docking difficulty levels, and classes of interactions including enzyme‐containing complexes, antibody–antigen complexes, as well as a range of other complex types. Comparison of AlphaFold performance with the performance of a global protein‐docking algorithm, ZDOCK^33^ showed remarkable and superior accuracy across the benchmark, even with only five models generated per test case. Determinants of modeling success were assessed by case category and other features, and a number of scoring functions, in addition to predicted TM‐score (pTM^34^ corresponding to overall topological accuracy) and predicted local difference distance test (pLDDT^35^ corresponding to local structural accuracy) scores generated by AlphaFold, were tested to find optimal scoring criteria to identify correct docking models from AlphaFold. We also tested a recently released version of AlphaFold, named AlphaFold‐Multimer, that was specifically trained to model protein–protein complexes.^36^ These results illustrate that while not successful for all cases and complex types, AlphaFold is a powerful tool for complex modeling, showing the power and advantage of end‐to‐end deep learning versus previous docking approaches. Our results also highlight areas for future optimization and developments in this framework, or other end‐to‐end deep learning frameworks, to effectively and reliably model most or all‐transient protein–protein complexes.

## 2 RESULTS

### 2.1 Performance of AlphaFold on protein–protein complex prediction

To assess the accuracy of AlphaFold in predicting structures of transient protein–protein complexes, we used Protein–Protein Docking Benchmark 5.5 (BM5.5),^20^, ^23^ which contains complexes spanning many classes of interactions that were identified from the Protein Data Bank^37^ using an automated pipeline followed by manual inspection and curation. All heterodimeric protein–protein complexes from that benchmark were identified for this analysis, corresponding to 152 test cases (Table S1). Based on levels of binding conformational changes and previously defined criteria,^38^ the cases had unbound docking difficulty classifications of Rigid (95 cases), Medium difficulty (34 cases) and Difficult (23 cases). Sequences of the two chains from each test case were input to AlphaFold, which generated structural models of the protein complexes using unpaired MSAs, without the use of templates. Additionally, the “advanced” interface in ColabFold^29^ was utilized to generate protein complex models using the AlphaFold framework. ColabFold uses different databases and a different MSA generation algorithm, but its speed and web accessibility make it a useful alternative to a locally installed full AlphaFold pipeline. To permit comparison with a current docking approach, the rigid‐body docking program ZDOCK (version 3.0.2)^33^ and the IRAD scoring function were used to perform global docking and rank models for all complexes, using unbound protein structures as input.

**FIGURE 1:**
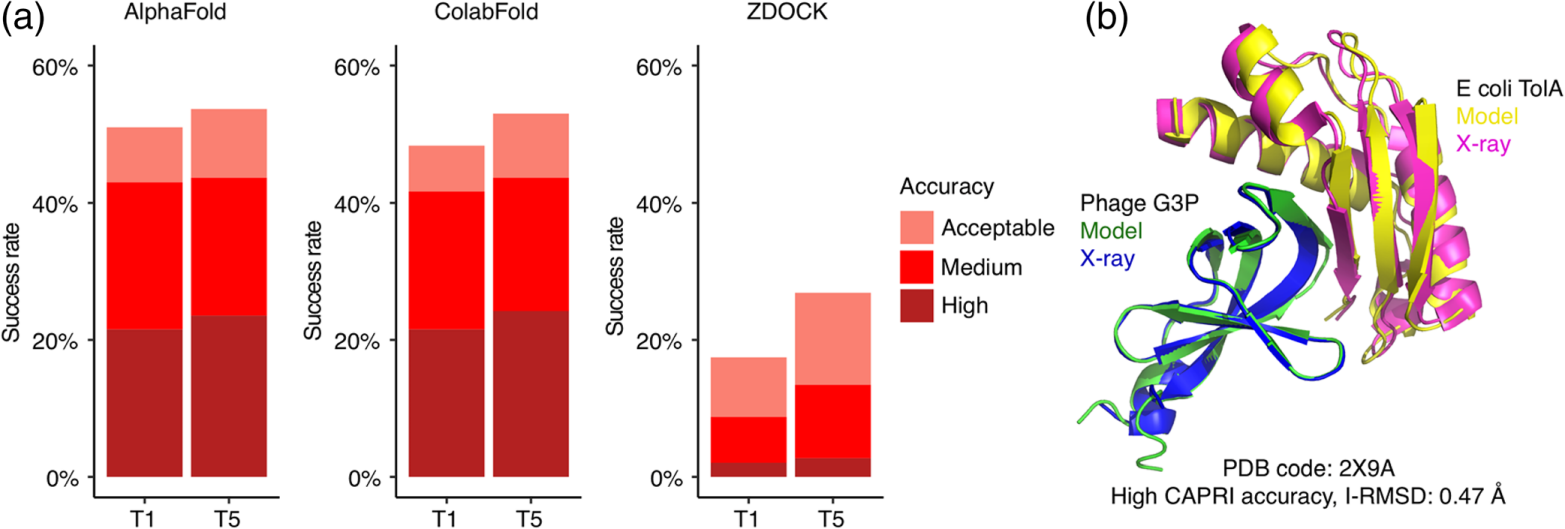
Transient protein–protein complex structure prediction success by AlphaFold, ColabFold and ZDOCK. End‐to‐end modeling using AlphaFold^24^ and ColabFold^29^ was performed on 152 complex test cases (details in Figure S1). AlphaFold failed to generate predictions for three complexes, thus AlphaFold predictions were obtained for 149 complexes; these 149 test cases were used to calculate success rates in this figure. Docking models were also generated with ZDOCK,^33^ using unbound protein structures as input. All sets of models were assessed for near‐native predictions using CAPRI criteria for high, medium, and acceptable accuracy. (a) Complex prediction success of AlphaFold, ColabFold, and ZDOCK for the top 1 (T1) and top 5 (T5) models considered. AlphaFold and ColabFold models were ranked by AlphaFold pTM scores, and ZDOCK models were ranked by IRAD scores.^39^ The percent success was calculated as the percentage of test cases with a given model accuracy from the top N models considered. Bars are colored according to the CAPRI quality classes. (b) Example of an accurately predicted complex structure (PDB code: 2X9A) by AlphaFold. This model has high accuracy by CAPRI criteria (I‐RMSD = 0.47) and has the highest pTM score (pTM = 0.77) of all five models generated for this complex. Structures are superposed by Phage G3P, with the model and the X‐ray structure chains are colored separately as indicated. For clarity, regions modeled by AlphaFold but unresolved in the X‐ray structure are not shown in the figure

The performance of AlphaFold, ColabFold, and ZDOCK was assessed by comparison of models with experimentally determined structures of the bound complexes; overall success rate comparisons are shown in Figure 1a, for top 1 (T1) and top 5 (T5) ranked models for each test case, with per‐case performance shown in Figure S1. Models were assessed as acceptable, medium, or high accuracy, or incorrect, based on using CAPRI criteria,^22^ which are based on comparison of models with corresponding experimentally determined structures using ligand root mean square distance (L‐RMSD), interface residue root mean square distance (I‐RMSD), and fraction of native interface residue contacts (*f*_nat_) metrics. While acceptable accuracy models can include moderate deviation from known structures (including models with up to 10 Å L‐RMSD), medium and high‐accuracy models are more reflective of previously utilized model accuracy cutoffs, such as the 2.5 Å I‐RMSD cutoff used for near‐native models by Chen and Weng^40^; accordingly, multiple studies have used the medium accuracy cutoff to identify near‐native models.^41^, ^42^ Remarkably, AlphaFold was able to generate models with acceptable or higher accuracy for approximately half (51%) of the 149 test cases for which models were generated, and for many of those cases, medium or better accuracy (43%) or high accuracy (21%) models were generated. Additionally, the top‐ranked model (T1) based on AlphaFold pTM score often represented the highest accuracy level for each case, and only a modest improvement in success was observed when allowing five predicted models per case (T5) (54%, 44%, and 23% success rates for acceptable accuracy or better, medium accuracy or better, or high accuracy, respectively). The success rate for ColabFold was similar to the success of AlphaFold, indicating that the different sequence databases and MSA procedure did not reduce or otherwise alter the capability of the AlphaFold deep learning model to generate near‐native complex models. Inspection of per‐case performance (Figure S1) confirmed that ColabFold and AlphaFold success was highly correlated across the test cases. Rigid‐body global docking success from ZDOCK was considerably lower than AlphaFold and ColabFold, particularly for medium and high accuracy models (13% acceptable or higher accuracy, 9% medium or higher accuracy, 1% high accuracy success for top‐ranked models), although a subset of cases was successful for ZDOCK while not successfully predicted by AlphaFold or ColabFold (Figure S1). A representative successfully modeled complex from AlphaFold is shown in comparison with the experimentally determined structure of the complex (PDB code 2X9A; *Escherichia coli* TolA/Phage G3B complex) in Figure 1b, demonstrating modeling of a virus‐host protein–protein interaction with atomic‐level accuracy. As that complex structure was released in 2010, it is possible that one or both of the component proteins were part of the AlphaFold training set, however the protein–protein interface and binding orientation were not.

### 2.2 Determinants of successful and unsuccessful AlphaFold performance

**FIGURE 2:**
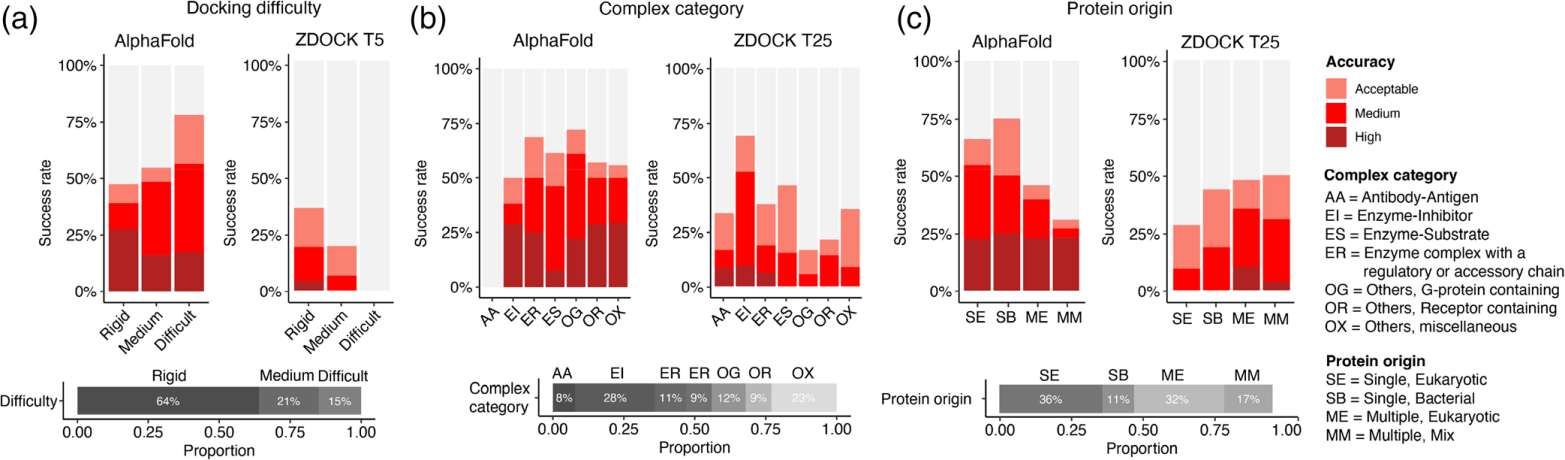
Determinants of successful performance. (a) Prediction success of AlphaFold and ZDOCK, grouped by docking difficulty. Based on binding conformational changes as defined by BM5.5,^20^, ^23^ cases are categorized into “rigid,” “medium,” and “difficult” docking difficulty levels. To evaluate the success rate, all five models from AlphaFold and top five ZDOCK models were considered. (b) Prediction success of AlphaFold and ZDOCK grouped by complex category. To evaluate the success rate, all five models from AlphaFold and top 25 ZDOCK models were considered. The number of ZDOCK models was increased to 25 to allow for sufficient success rates to show its relative performance among the categories. (c) Prediction success of AlphaFold and ZDOCK grouped by protein source organism(s). To evaluate the success rate, all five models from AlphaFold and top 25 ZDOCK models were considered. Based on the source of subunit proteins in the complex structures, each case is classified as either “single, eukaryotic” (SE, denoting proteins from the same eukaryotic organism), “single, bacterial” (SB, denoting proteins from the same bacterial organism), “multiple, eukaryotic” (ME, denoting proteins from different eukaryotic organisms), or “multiple, mix” (MM, denoting proteins from mixed origins). Two additional protein source classes, corresponding to proteins from different bacterial organisms, and proteins of viral origin, were omitted from the success plot due to limited representation in each category (four and two cases in those classes, respectively). Bars are colored by model accuracy as indicated in (a). The horizontal stacked bars below each success rate plot denote the composition of the categories by class

To investigate the determinants of successful performance for AlphaFold, we compared performance across subsets of cases divided by various biological and structural properties (Figure 2). As expected, previously assigned test case difficulty classifications, which are based on binding conformational change between unbound and bound structures,^20^, ^23^ did not markedly impact the success of AlphaFold; for acceptable or higher accuracy predictions in the set of five models, success rates for AlphaFold were found to be 47%, 55%, and 78% for rigid, medium, and difficult docking difficulty categories, respectively (Figure 2a). The increase in AlphaFold success for cases in the Difficult docking case category relative to the other two categories was less pronounced or not observed for more stringent model accuracy criteria of medium and high accuracy. AlphaFold medium or higher model accuracy success rates for the difficulty categories were 39% (rigid), 48% (medium), and 57% (difficult), while high accuracy model success rates were 27% (rigid), 16% (medium), and 17% (difficult). For the docking algorithm ZDOCK, which unlike AlphaFold used unbound protein structures as input, success rates for the top 5 ranked models were 36% (rigid), 19% (medium), and 0% (difficult) for acceptable or higher accuracy models. This reduced success of ZDOCK for progressively higher docking difficulty categories is in accordance with previous benchmarking studies with ZDOCK and other methods that use unbound structures as input.^20^, ^23^ While the “fold‐and‐dock” approach in AlphaFold is likely at least partly responsible for improved modeling context‐specific conformations versus the reliance of unbound structures for rigid‐body docking, it remains possible, as noted above, that some bound conformations of individual protein components in B5.5 are part of the AlphaFold training set, which would provide an additional advantage for AlphaFold versus the use of the unbound structures, or models of unbound structures, as input for complex assembly.

Performance across benchmark cases was also assessed by complex category, as well as protein source (Figure 2b,c). Notably, the antibody–antigen complexes had no successfully generated models, while other complex categories considered all showed approximately commensurate levels of AlphaFold performance. There was no major difference observed in AlphaFold success for prediction of complexes with proteins from eukaryotic or bacterial organisms, and while there was a slight reduction in overall success when the two proteins in a complex came from different organisms (which theoretically could impact a cross‐interface signal of an MSA), the success for high quality models was approximately the same (~25%) regardless of single versus multiple source organism, or source organism type.

**FIGURE 3:**
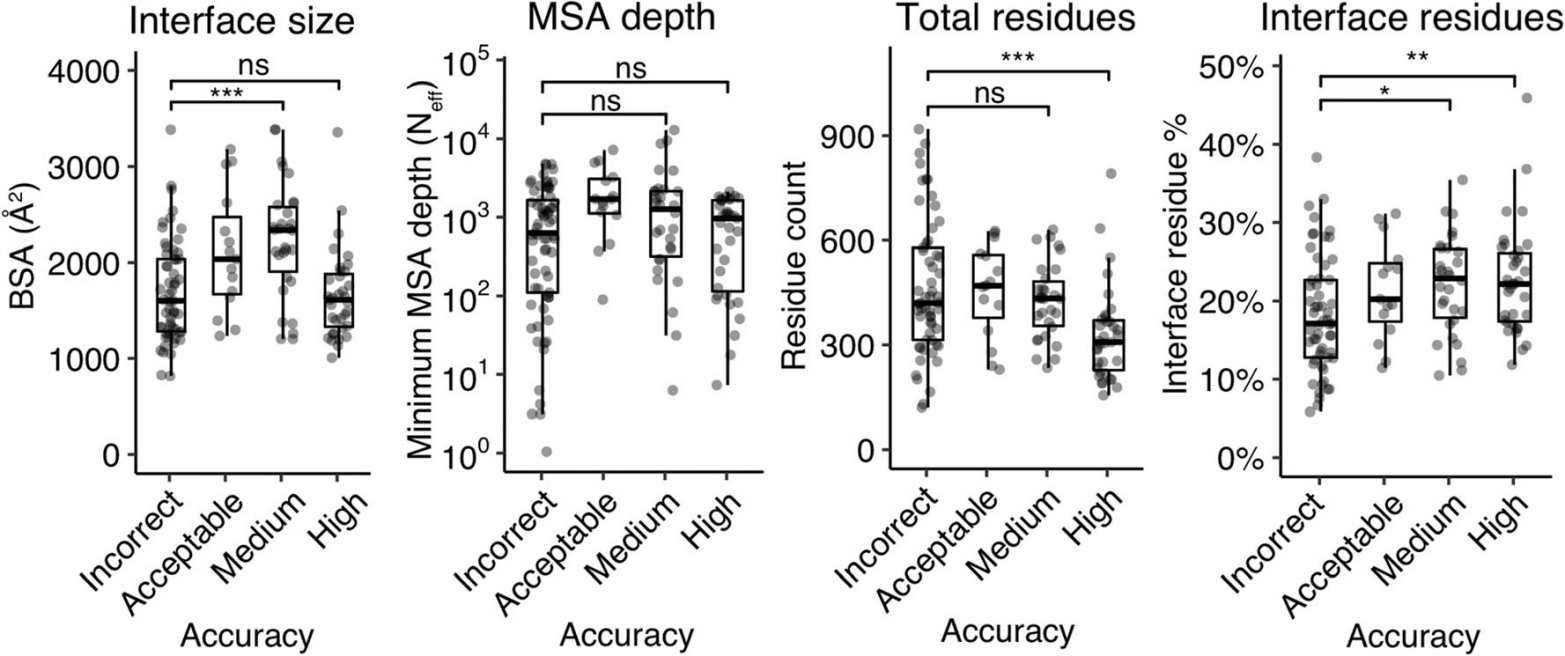
Assessing test case features associated with AlphaFold success. Protein complex and MSA feature values were computed for all cases, which are shown according to AlphaFold success (best AlphaFold model accuracy in the five models for that case). Features shown are interface buried surface area (BSA), MSA depth (*N*_eff_) for the ligand or receptor (minimum value of the two), total number of residues, and percent of total residues in the protein–protein interface. Statistical significance values (Wilcoxon rank‐sum test) were calculated between feature values for sets of cases with incorrect versus medium and incorrect versus high‐ CAPRI accuracy, as noted at top (ns: *p* > .05, *p ≤ .05, **p ≤ .01, ***p ≤ .001)

We performed analysis of a series of geometric and other protein complex properties to identify possible relationships with AlphaFold modeling success. Computed interface features were assessed for association with incorrectly modeled cases versus cases with near‐native AlphaFold complex models (medium and/or high CAPRI accuracy) (Figure 3, Table S2). Greater interface size, measured by buried surface area (BSA), was found to be associated with AlphaFold success for incorrect versus medium/high‐accuracy cases (*p* = .007; Table S2), and incorrect versus medium accuracy cases (*p* ≤ .001; Figure 3), yet this trend was not observed when comparing incorrect versus high accuracy cases. To account for possible bias from antibody–antigen features and their pronounced lack of AlphaFold success noted above, comparisons were made with antibody–antigen cases excluded (Figure S2), yielding essentially the same results as with all cases (Figure 3). Limited MSA depth for either or both partner proteins was explored as a possible factor in poor predictive performance, but it was not found to have a significant impact (Figure 3). Among the case features analyzed, we found that larger protein sizes, and a relatively small interface in comparison to protein size (measured either by number of residues or solvent accessible surface area), were most associated with poor complex modeling performance (Figure 3, Table S2).

We also explored the accuracy of individual chain structural modeling and MSA depth (Figure S3); while a range of chain alignment depths (number of effective sequences [*N*_eff_]) were observed, in most cases the individual ligand and receptor chains were modeled accurately (backbone RMSD with bound component chains <2.5 Å). Protein complex model accuracies based on interface residue RMSD (I‐RMSD) and CAPRI criteria did not show a relationship with maximum subunit chain RMSD (Figure S3c), indicating that incorrect binding mode, versus inaccurate chain folding, was the primary cause of incorrect AlphaFold complex models. AlphaFold models representing incorrect binding mode and inaccurate chain folding are shown in Figure S3d and Figure S3e, respectively.

### 2.3 Impact of alternative AlphaFold parameters and input

**FIGURE 4:**
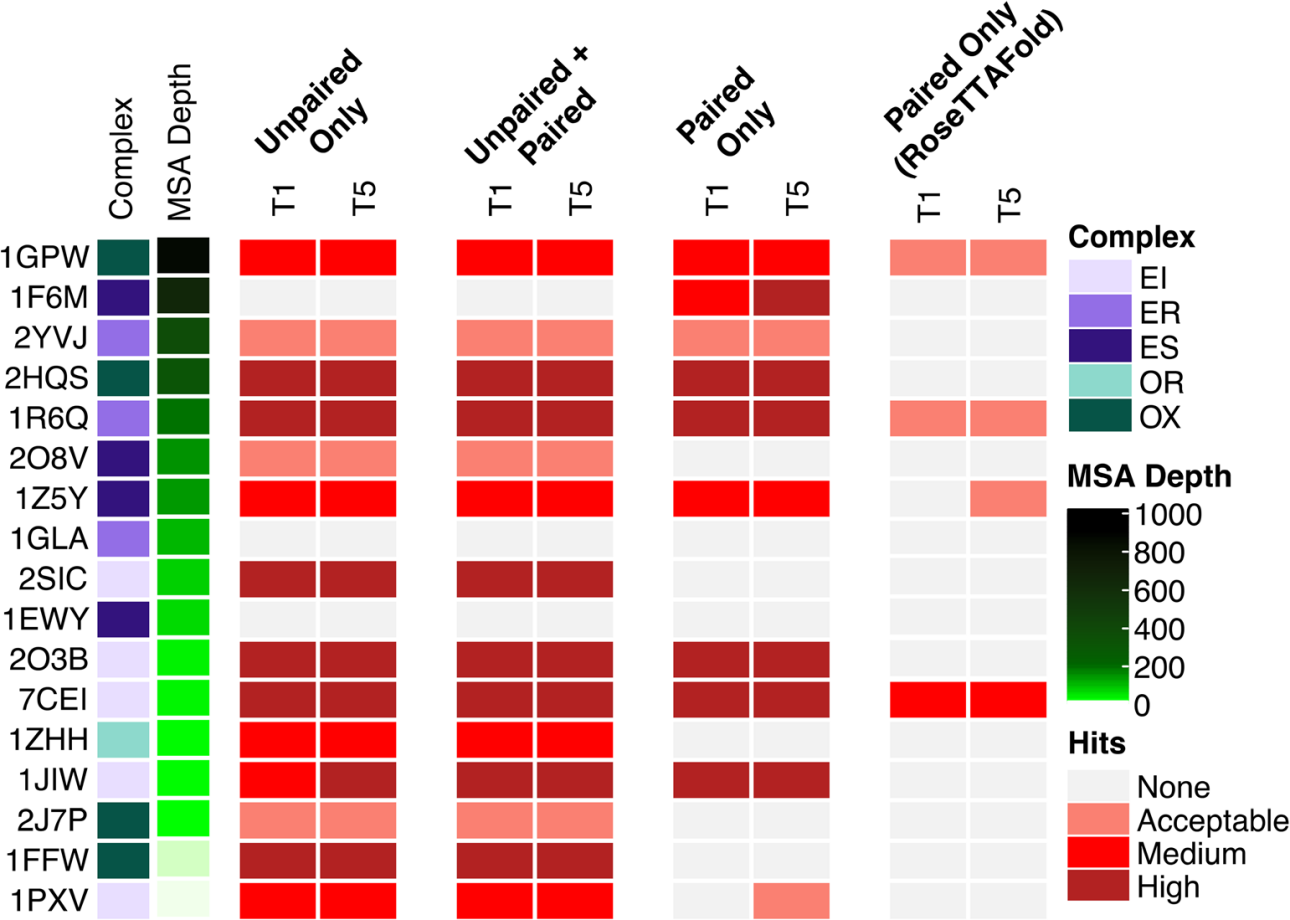
The impact of MSA pairing on prediction accuracy. MSAs were generated using MMseqs2 using the “advanced” interface of ColabFold.^29^ Pairing was performed in ColabFold on a total of 17 cases whose ligand and receptor proteins come from the same prokaryotic organism. Cases in the heatmap were sorted by the paired MSA depth (*N*_eff_; see Material and Methods for details) from the largest to the smallest values. Structural predictions were generated with the “advanced” interface of ColabFold, and RoseTTAFold^28^ (through the Robetta server). All models were assessed for near‐native predictions within the top‐ranked (T1) and top 5 (T5) models using CAPRI criteria. Complex category: Enzyme‐inhibitor (EI), enzyme complex with a regulatory or accessory chain (ER), enzyme‐substrate (ES), others, receptor‐containing (OR); others, miscellaneous (OX)

Given the success of AlphaFold with unpaired MSAs, consisting of individual MSAs for each protein, we tested the impact of the use of paired sequences, which represent both chains as a single sequence in the MSA, within the input MSAs. Due to its capability to provide a coevolution signal between protein residues across an interface, which can then be inferred as cross‐interface contacts, use of paired sequences in MSAs has shown promise previously for protein complex structure prediction.^28^, ^43^, ^44^ MSAs with paired sequences were obtained from the ColabFold Google Colab site (on September 4, 2021);^29^ these sequence pairs were generated with an automated algorithm intended for prokaryotic proteins, thus a set of 17 cases from BM5.5 was tested that contain two prokaryotic proteins from the same organism. As shown in Figure 4, the addition of paired sequences did not appear to improve AlphaFold performance over use of unpaired MSAs as input, while use of paired sequences alone was detrimental to successful complex modeling in some cases. One notable exception was test case 1F6M, which had a relatively high number of paired sequences in the MSA. When paired sequences alone were used, high accuracy models were obtained for test case 1F6M, whereas no hits were obtained when unpaired sequences were included. For comparison, the same paired‐only MSAs were input to RoseTTAFold,^28^ which according to its authors can utilize paired MSAs to predict complex structures; while some accurate models were obtained, we observed lower overall success for the models generated with that method.

We separately compared the use of paired sequences, unpaired sequences, or both as MSA input for AlphaFold‐Multimer,^36^ which was trained specifically to model protein–protein complexes (Figure S4). The paired‐only results showed accuracy improvements in some cases versus the unpaired‐only baseline, as well as unpaired + paired inputs (e.g., 1F6M, 1ZHH), while loss of near‐native models for paired‐only was observed for two cases with very low‐paired MSA depths (1FFW, 1PXV). Thus it seems possible that AlphaFold‐Multimer can better utilize paired‐only inputs (with sufficient sequences) for complex modeling than AlphaFold, however it should be noted that the overlap of the set of complexes in this test set with the AlphaFold‐Multimer training set (both interfaces and component proteins) may mask comparative differences among MSA inputs and likely leads to high overall baseline performance in Figure S4.

We also tested altered parameters for the number of iterative refinement cycles (*N*_cycle_) and MSA ensemble size (*N*_ensemble_) in AlphaFold, for a subset of the docking test cases selected to represent the antibody, enzyme, and “other” protein complex types, and observed very little effect on predictive performance (Figure S5).

### 2.4 Docking model discrimination by scoring metrics

**FIGURE 5:**
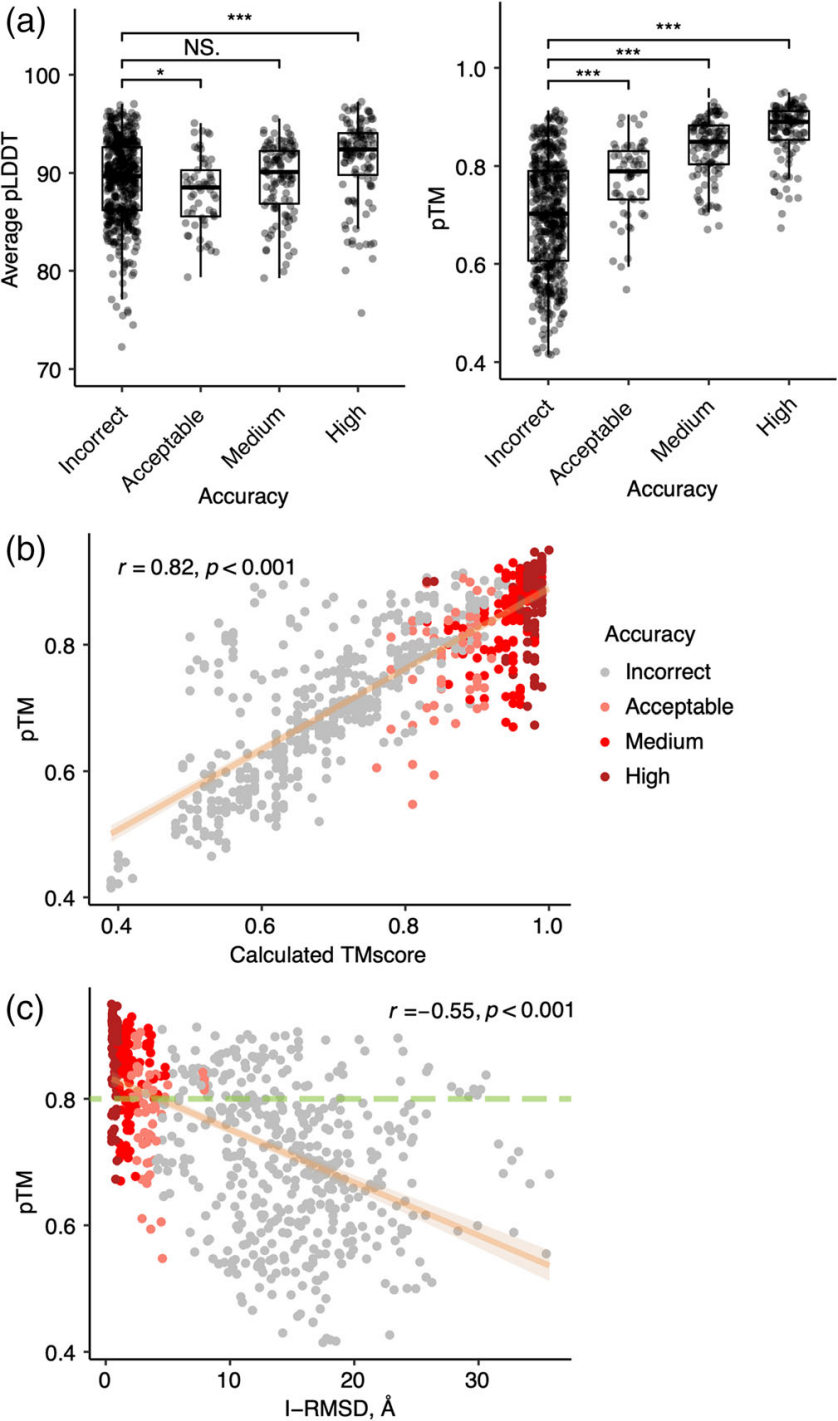
Association between AlphaFold predicted scores and docking model quality. (a) Average pLDDT and pTM per CAPRI criteria. Statistical significance (Wilcoxon rank‐sum test) between average pLDDT or pTM of incorrect versus acceptable, incorrect versus medium and incorrect versus high CAPRI criteria is indicated at the top (ns: *p* > .05, *p ≤ .05, **p ≤ .01, ***p ≤ .001). (b) Comparisons between pTM and calculated TM‐score and (c) between pTM and I‐RMSD are shown as scatter plots. All 5 models for 149 cases are shown as points, colored by model quality by CAPRI criteria. Linear regression is shown along with the 95% confidence interval (orange area), and Pearson's correlation coefficients and correlation p‐values are denoted in (b) and (c). In (c), the dashed green line indicates a possible pTM score cutoff (pTM = 0.8) for selection of accurate docking models, based on optimization of sensitivity and specificity for incorrect versus medium and high model discrimination

Given the reported success of AlphaFold in predicting the quality of its monomeric protein models through scores representing local accuracy (pLDDT) and global accuracy (pTM),^24^ we tested the discriminative capabilities of these values in the context of protein complex modeling (Figure 5). Average pLDDT scores and pTM scores for AlphaFold complex models were both found to discriminate incorrect versus higher model accuracy classifications, with pTM scores performing moderately better (Figure 5a). Comparison of pTM with complex model TM‐scores^34^ showed a relatively strong correlation of the predicted with the calculated accuracy value (*r* = .82; *p* < .001; Figure 5b), while pTM exhibited a significant, though moderately weaker, correlation with I‐RMSD of AlphaFold models (*r* = −.55, *p* < .001; Figure 5c).

**FIGURE 6:**
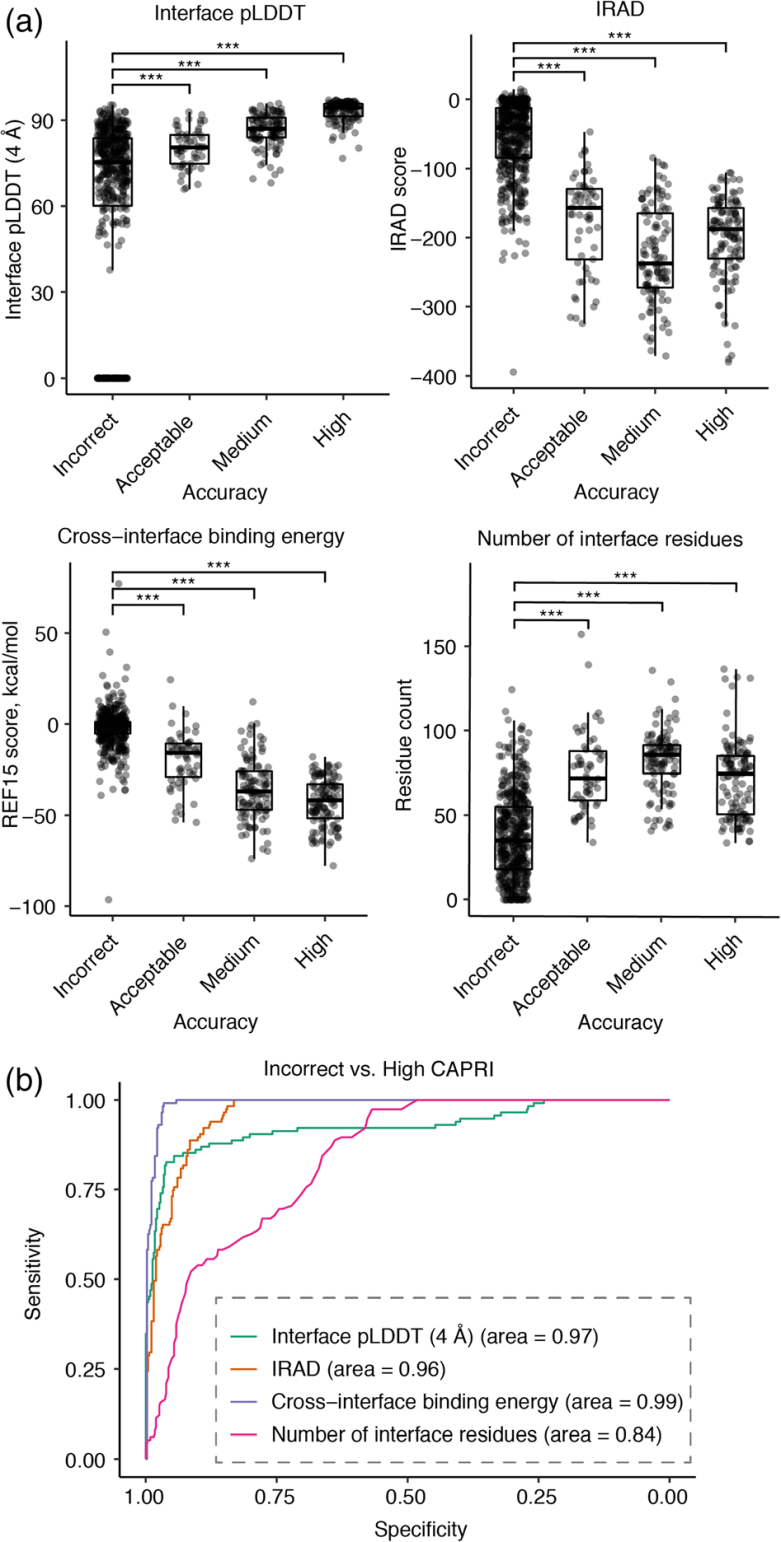
Association between alternative scoring metrics and docking model quality. (a) Distributions of interface pLDDT (4 Å), IRAD, Rosetta cross‐interface binding energy, and number of interface residues for AlphaFold models grouped by CAPRI criteria. An interface pLDDT score of 0 was assigned to models without any interface contacts within the distance cutoff (4 Å). Constrained local minimization was performed using Rosetta FastRelax^65^ to resolve unfavorable local geometries or clashes in models, and post‐relaxation models were scored with IRAD and the Rosetta^45^ InterfaceAnalyzer protocol, with the latter used to calculate cross‐interface binding energy scores (based on the Rosetta REF15 energy function^66^) and the number of interface residues. Statistical significance values (Wilcoxon rank‐sum test) between scores of incorrect versus acceptable, incorrect versus medium, incorrect versus high CAPRI criteria are indicated at the top of each plot (***p ≤ .001). Each point corresponds to one AlphaFold model, and all five AlphaFold models for 149 test cases are represented. (b) ROC curves among the scoring metrics for classifying incorrect versus high accuracy models by CAPRI criteria, with corresponding AUC values denoted in parentheses

While pTM and pLDDT showed some capability to identify correct versus incorrect complex structural models, the overlap in scores between accuracy categories (Figure 5a) led us to explore additional scoring functions to predict the structural quality of AlphaFold models (Figure 6, Table 1). Given the likely importance of interface residue contacts and packing, versus the folding accuracy of interface‐distal protein regions, in discrimination of correct versus incorrect docking models, we tested two residue‐level predicted accuracy metrics from AlphaFold, PAE (Predicted Aligned Error, corresponding to expected error in the position of one residue with respect to another residue in a model)^44^ and pLDDT, for predicted protein–protein interface residues alone, to assess model discrimination capabilities. Alternative formulations of these metrics were tested with more permissive interface definitions, versus the originally tested 4 Å interface cutoff, but no major difference in model assessment accuracy was observed (Figure S6, Table S3). Interface PAE and interface pLDDT values showed major improvement compared with average pLDDT and pTM from AlphaFold in discriminating accurate complex models, based on receiver operating characteristic area under the curve (AUC) metrics (Table 1), particularly for the discrimination of models in the most populous and divergent incorrect and high model accuracy categories (AUCs of 0.93 and 0.97 for interface PAE and interface pLDDT, respectively). Relatively high‐AUC values were also observed for previously reported docking model ranking methods ZRANK2^15^ and IRAD^29^ (Table 1), while an interface energy score from Rosetta^45^ (cross‐interface binding energy) resulted in the highest model classification accuracy, based on the binary classification AUC metrics (Table 1). However, estimated 95% confidence intervals (95% CI) (included in Table 1) showed overlap between AUC value ranges for ZRANK2, IRAD, and Rosetta cross‐interface binding energy for incorrect versus medium or high accuracy models, indicating that their performance is essentially equivalent for that model accuracy discrimination. Based on discrimination of incorrect versus medium and high accuracy models and maximization of sensitivity and sensitivity, possible score cutoffs for model selection are pTM = 0.8 (shown as dashed line in Figure 5c), interface pLDDT = 84, IRAD = −128, and Rosetta cross‐interface binding energy = −16. While performing lower than the Rosetta binding energy score, some relatively simple protein interface assessments, such as the number of interface hydrogen bonds, showed some capability to classify the accuracy of AlphaFold models.

**TABLE 1:** Area under the ROC curve (AUC) values and 95% confidence intervals (shown in parentheses) for protein quality classes as a function of different scoring metrics

Score^a^	Binary classification^b^		Multiclass classification^c^
	Incorrect vs. high	Incorrect vs. medium and high	
Average pLDDT	0.66 (0.60–0.72)	0.59 (0.54–0.63)	0.64 (0.58–0.67)
Average resolved pLDDT	0.81 (0.76–0.85)	0.69 (0.64–0.72)	0.69 (0.65–0.73)
pTM	0.92 (0.89–0.95)	0.89 (0.86–0.91)	0.80 (0.76–0.82)
Interface PAE (4 Å)	0.93 (0.89–0.96)	0.90 (0.87–0.92)	0.81 (0.77–0.83)
Interface pLDDT (4 Å)	0.97 (0.95–0.98)	0.90 (0.87–0.92)	0.84 (0.82–0.85)
IRAD	0.96 (0.95–0.98)	0.97 (0.95–0.97)	0.80 (0.76–0.82)
ZRANK	0.95 (0.93–0.96)	0.95 (0.94–0.96)	0.77 (0.73–0.79)
Cross‐interface binding energy	0.99 (0.99–1.00)	0.97 (0.95–0.98)	0.84 (0.81–0.86)
Interface area	0.90 (0.88–0.93)	0.92 (0.90–0.94)	0.77 (0.73–0.79)
Number of interface hydrogen bonds	0.96 (0.95–0.98)	0.94 (0.92–0.95)	0.81 (0.78–0.83)
Number of interface residues	0.84 (0.80–0.87)	0.87 (0.84–0.89)	0.73 (0.66–0.74)
Shape complementarity	0.91 (0.88 to 0.93)	0.85 (0.81–0.87)	0.79 (0.76–0.81)

^a^ Scoring methods. “average resolved pLDDT”: average pLDDT on the resolved region, “interface PAE (4 Å)”: average PAE of pairs of interface residues within 4 Å distance cutoff, “interface pLDDT (4 Å)”: average pLDDT of interface residues within 4 Å distance cutoff. “cross‐interface binding energy,” “interface area,” “number of interface hydrogen bonds,” “number of interface residues” and “shape complementarity” were calculated using the Rosetta InterfaceAnalyzer (see Methods for details).

^b^ The AUC values of the binary classification were calculated using the pROC package^73^ in R. The 95% confidence intervals were calculated by pROC.

^c^ The AUC values of the multi‐class classification were calculated with multiROC package^74^, ^75^ in R. The 95% confidence intervals of multi‐class AUC values were calculated with the boot package^72^, ^73^ in R with adjusted bootstrap percentile (BCa) interval.

### 2.5 Expanded antibody–antigen complex benchmarking

**FIGURE 7:**
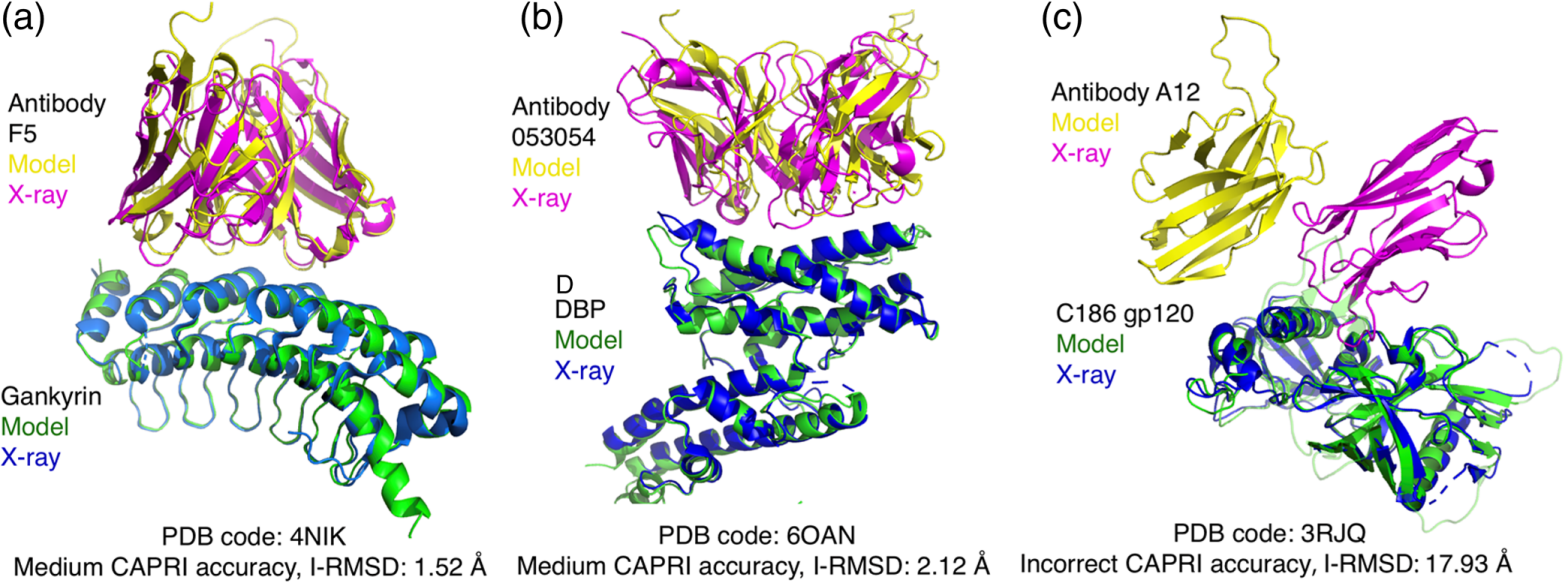
Examples of antibody–antigen complex structure predictions by AlphaFold. (a) Native and top‐ranked AlphaFold model (pTM = 0.78) for PDB 4NIK (F5 antibody/human gankyrin complex). This model is of medium accuracy by CAPRI criteria (I‐RMSD = 1.52 Å). Modeled and X‐ray complex structures are colored as indicated and shown superposed by gankryin. Unresolved regions modeled by AlphaFold are not shown. (b) Native and top‐ranked AlphaFold model (pTM = 0.61) for PDB 6OAN (053054 antibody/P vivax DBP complex). This model is of medium accuracy by CAPRI criteria (I‐RMSD = 2.12 Å). Modeled and X‐ray structures are colored as indicated, shown superposed by DBP, and unresolved regions modeled by AlphaFold are not shown. (c) Native and top‐ranked AlphaFold model (pTM = 0.66) for PDB 3RJQ (A12 nanobody/HIV C186 gp120 complex), superposed by C186 gp120. This AlphaFold model does not have contacting residues between the proteins within a 5 Å distance cutoff. Structures are colored as indicated in the figure, and unresolved regions modeled by AlphaFold on C186 gp120 are shown in light green

Due to the lack of any successful structural prediction of 11 antibody–antigen complexes from the BM5.5 set, we assembled a set of 20 additional nonredundant antibody–antigen complexes with known structures to assess AlphaFold accuracy (Table S4). These complexes include a variety of antigens, and as the BM5.5 heterodimer set included only nanobodies, a number of single‐chain antibodies with both heavy and light chains represented were selected for the additional cases (comprising 17 out of 20 of the cases), while the remaining three cases include single‐domain nanobodies. While AlphaFold modeling of most of those complex structures resulted in no accurate predictions, surprisingly two of the antibody–antigen complexes were modeled accurately, with medium CAPRI accuracy models ranked #1 for each complex (Table S4, with models shown in Figure 7a,b).

Inspection of AlphaFold models of antibody–antigen complexes indicated that many of the inaccurate models had few or no contacts between antibody and antigen chains; one example is shown in Figure 7c). Indeed, analysis of the percentage of models with no atomic contacts between chains showed that antibody–antigen cases had relatively high rates of such models in comparison with the other protein complex categories in the benchmark (Figure S7).

### 2.6 AlphaFold performance for non‐immunoglobulin antibody–antigen complexes

After confirming the limited success of AlphaFold in predicting antibody–antigen complex structures, we performed additional modeling assessments in AlphaFold to identify factors responsible for that performance. While smaller interface size and larger complex structure size were found to be associated with lower AlphaFold success for the overall set of cases (Figure 3, Table S2), additional features specific to antibody–antigen complex structures or sequences likely reduce AlphaFold performance for that class. To assess whether the immunoglobulin architecture shared by the antibodies impacted AlphaFold performance, we modeled a set of complexes containing nonimmunoglobulin receptors in complex with protein targets using AlphaFold (Table S5). These receptors correspond to variable lymphocyte receptors (VLRs), which are adaptive immune receptors found in jawless vertebrates (e.g., sea lampreys), and recognize protein and nonprotein antigens with leucine‐rich repeat architectures.^47^ Three complex structures with VLR‐based receptors, referred to as repebodies,^48^ were also included in this set of cases. Only one out of the seven VLR and repebody complexes tested had any correct models from AlphaFold (Table S5), indicating that the immunoglobulin architecture was not responsible for the observed limited AlphaFold success for antibody–antigen complexes.

### 2.7 AlphaFold‐Multimer performance for antibody–antigen and T cell receptor complex modeling

After observing limited success of AlphaFold for modeling of antibody–antigen complexes, we tested modeling of that class of complexes with AlphaFold‐Multimer.^36^ As AlphaFold‐Multimer training included protein–protein interfaces from structures released before May 2018,^36^ the test set included only antibody–antigen complexes from May 2018‐present that are not redundant with pre‐May 2018 structures, generated as part of an update to our recently reported set of antibody–antigen docking test cases.^23^ Additionally, to investigate the impact of MSAs on antibody–antigen performance, we modeled the antibody–antigen complex structures with and without MSA input. In this context, we allowed the use of structural templates for each chain, in order to focus on complex modeling accuracy without reduction of tertiary structure fidelity due to the lack of MSAs. Out of seven antibody–antigen complexes in the test set (Table 2), one complex (6U54) contains a nanobody. For comparison, recently released nonantibody complex structures from the “Benchmark 2” set described by Ghani et al.^49^ were also tested. ColabFold was used to run AlphaFold‐Multimer for these cases, due to its previously observed performance commensurate with full AlphaFold (Figure 1, Table S1), and the capability to remove MSA input features (noted in Methods).

**TABLE 2:** AlphaFold‐Multimer performance for recently released antibody–antigen and non‐antibody complex structures, with and without multiple sequence alignment input

		With MSA^a^		No MSA^a^	
Set	Case	T1	T5	T1	T5
Antibody–antigen complexes	6A4K	Incorrect	Incorrect	Incorrect	Incorrect
	6HX4	Medium	Medium	Incorrect	Incorrect
	6P50	Incorrect	Incorrect	Incorrect	Incorrect
	6PXH	Incorrect	Incorrect	Incorrect	Incorrect
	6Q0O	Acceptable	Acceptable	Incorrect	Incorrect
	6U54	Medium	Medium	Medium	Medium
	6ZTR	Incorrect	Incorrect	Incorrect	Incorrect
Other complexes (non‐antibody) from Ghani et al.^49^	5ZNG	Incorrect	Incorrect	Incorrect	Incorrect
	6A6I	Acceptable	Acceptable	Incorrect	Incorrect
	6GS2	Medium	Medium	Incorrect	Incorrect
	6H4B	Medium	High	Incorrect	Incorrect
	6IF2	Medium	Medium	Incorrect	Incorrect
	6II6	High	High	Incorrect	Incorrect
	6ONO	Medium	Medium	Incorrect	Incorrect
	6PNQ	Incorrect	Incorrect	Incorrect	Incorrect
	6Q76	High	High	High	High
	6U08	High	High	Incorrect	Incorrect
	6ZBK	High	High	Acceptable	Acceptable
	7AYE	High	High	Acceptable	Acceptable
	7D2T	High	High	Incorrect	Incorrect
	7M5F	Medium	Medium	Incorrect	Incorrect
	7 N10	High	High	Incorrect	Incorrect
	7NLJ	Incorrect	Incorrect	Incorrect	Incorrect
	7P8K	Medium	Medium	Incorrect	Incorrect

^a^ Modeling was performed using AlphaFold‐Multimer^36^ in ColabFold,^29^ with multiple sequence alignment (“With MSA”) or without multiple sequence alignment (single sequence, “No MSA”) feature input. Shown are CAPRI model accuracy levels for top‐ranked model (T1) and five models (T5) for each case, with medium and high accuracy levels highlighted with light red and dark red cell shading, respectively. Structural templates for subunits were enabled for all runs, to allow for accurate modeling of individual chains in the absence of MSAs, with a date cutoff of 4/30/2018 to avoid use of the bound complex subunit structures as templates.

Results from this assessment, shown in Table 2, highlight a major difference in overall predictive success for standard AlphaFold‐Multimer (with MSA input) between non‐antibody success (13/17 cases, or 76%, with a medium/high accuracy model ranked #1) versus antibody–antigen case success (2/7 cases, or 29%, with a medium/high accuracy model ranked #1). Furthermore, our results indicate that while the lack of MSA input and corresponding residue coevolutionary signal has a pronounced impact on near‐native accuracy (CAPRI medium/high models) for non‐antibody complexes, it appears to have less of impact on antibody–antigen complex structure prediction. Additional AlphaFold‐Multimer modeling of the same set of antibody–antigen complexes with no subunit templates and with MSA input did not affect predictive performance (Table S6). Taken together with the results for the non‐immunoglobulin (VLR) cases, it appears that the limited success of antibody–antigen complex modeling AlphaFold and AlphaFold‐Multimer is largely due to the lack of coevolution signal, demonstrated by the lack of effect of MSA input, versus structural or geometric features of those interfaces. Of relevance, others have recently noted the importance of MSAs and coevolution signals in AlphaFold's global conformational search.^50^ As the AlphaFold‐Multimer algorithm generates an interface pTM score (ipTM) which is used in conjunction with pTM to compute model scores,^36^ we examined the use of ipTM score alone in model accuracy discrimination for models from the set of cases in Table 2, and ipTM alone showed promising model discrimination accuracy, with an ipTM score threshold of approximately 0.75 corresponding to a possible model confidence cutoff (Figure S8).

**FIGURE 8:**
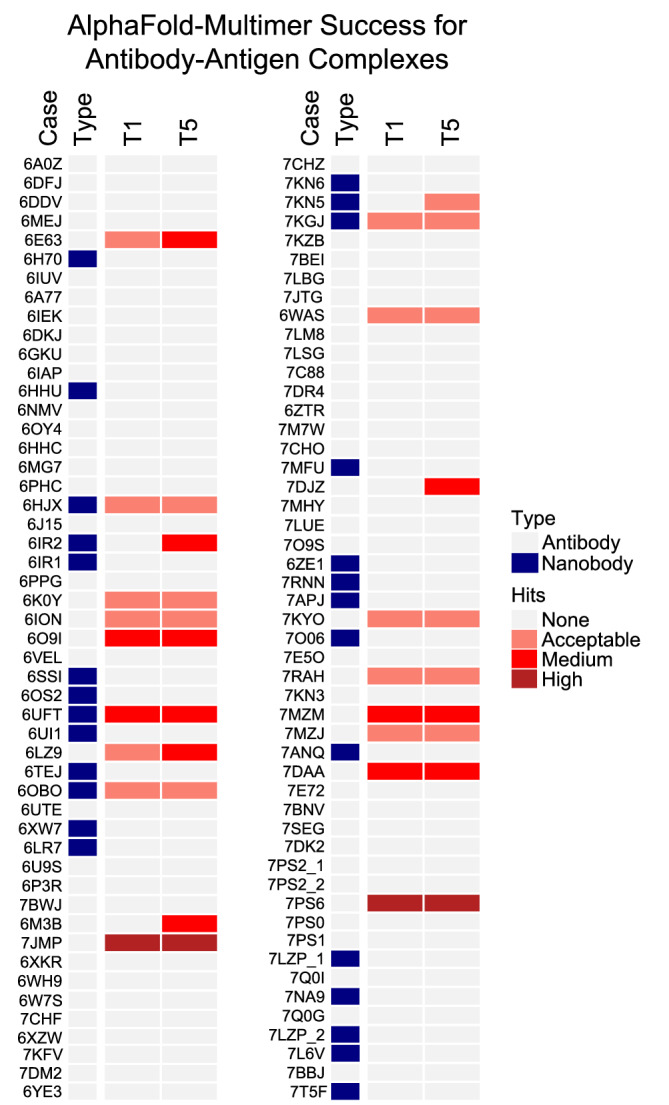
AlphaFold‐Multimer modeling success for an expanded set of recently released antibody–antigen complex structures. A benchmark set of 100 recently released antibody–antigen complex structures was modeled with AlphaFold‐Multimer, and all five models from AlphaFold‐Multimer per test case were assessed for accuracy using CAPRI criteria. AlphaFold‐Multimer was run in ColabFold with MSA input and with the use of structural templates released before April 30, 2018, and models were ranked by pTM score. Success for top 1 and top 5 (T1, T5) ranked predictions is shown, colored by CAPRI model accuracy as indicated in the key on right (Hits). “Type” distinguishes complexes containing heavy‐light chain antibodies (“Antibody”) and single‐chain nanobodies (“Nanobody”).

Due to the relatively limited number of antibody–antigen cases tested initially with AlphaFold‐Multimer (Table 2), we assembled a larger set of 100 recently released antibody–antigen complex structures for benchmarking AlphaFold‐Multimer for predictive performance with that class of complexes. All of the structures were recently released (May 2018 or later), and they contain complexes with heavy‐light chain antibodies (73 complexes) and nanobodies (27 complexes) (Table S7). In order to model the large number of cases, all complexes were modeled with AlphaFold‐Multimer in ColabFold, due to its effciency and comparable success to that of the full AlphaFold pipeline (Figure 1, Figure S1). Success rates for this set were found to be low, with 6% of cases with medium or high accuracy models ranked #1, and 11% of cases with medium or high accuracy models in one or more of the five models generated per case (Figure 8). This is in accordance with the limited success for antibody–antigen complex modeling briefly noted by the AlphaFold‐Multimer developers,^36^ and is similar to the success observed for the non‐antibody–antigen cases noted above without MSA input (1 out of 17 cases, or 6% success; Table 2).

Having determined the predictive performance of AlphaFold‐Multimer for antibody–antigen complexes, we tested that algorithm for its capability to model T cell receptor‐peptide–major histocompatibility complex (TCR‐pMHC) structures, to further delineate its modeling accuracy for adaptive immune recognition. Although most TCRs share a general binding site and orientation over the pMHC,^50^ their diversity of pMHC recognition modes, mediated by flexible and variable complementarity determining region loops, pose a challenge for predictive modeling methods, of which several have been developed based on docking^41^ and template‐based assembly.^51^, ^52^ We assembled a set of 14 Class I TCR‐pMHC complexes with known structures that were released in May 2018 or later, and modeling of those complexes with AlphaFold‐Multimer showed a success rate of 2 out of 14 complexes (14%) with near‐native (medium or high‐CAPRI accuracy) ranked at #1 or within the five models for each case (Table S8). This highlights another class of complexes that is challenging for the current implementation of AlphaFold‐Multimer, likely in part due to the limited coevolution signal in the interface. While there is evidence that TCR genes have co‐evolved with MHC genes to promote TCR‐pMHC interactions,^53^ the critical peptide–MHC and TCR‐peptide interfaces in TCR‐pMHC complexes are not guided by coevolution, and the accurate modeling of the bound peptide as well as the correctly docked TCR presents a clear challenge in a fold‐and‐dock scenario.

## 3 DISCUSSION

Our extensive testing of AlphaFold performance on a nonredundant benchmark of protein–protein complexes indicates that AlphaFold is largely successful at predicting binary transient protein complex structures. However, some complexes were not successfully modeled, most notably antibody–antigen and other adaptive immune interactions, while other categories of test cases showed upper limits of success as well. The limited number of antibody–antigen complex structures that were successfully modeled show that antibody complex modeling can be performed in some cases with the AlphaFold framework, while many of the incorrect models seem to be readily identifiable based on AlphaFold metrics of predicted interface residues, or previously developed energy‐based scoring functions.

While protein complex and interface size showed some associations with AlphaFold success for complexes in general, we found that the lack of useful coevolution signals in the MSAs for antibody–antigen complexes was likely responsible for the limited success of those cases, as shown by the lack of effect on performance when removing the MSA input signal. We tested this using a recently optimized version of AlphaFold for protein–protein interfaces, named AlphaFold‐Multimer,^36^ and the antibody–antigen modeling success with that version of AlphaFold was reflective of our results with the previous AlphaFold release (AlphaFold2),^24^ and in accordance with the observation that AlphaFold‐Multimer is “generally not able to predict” antibody–antigen complex structures noted by the AlphaFold‐Multimer authors.^36^ While some antibody–antigen interfaces have been reported to undergo coevolution in vivo, in particular with evolving viral antigens,^53^, ^54^ it is unlikely that corresponding sets of sequences are available for many antibody–antigen pairs in the AlphaFold and ColabFold sequence databases, or in general. However, as noted above, the geometric and structural elements of the AlphaFold and AlphaFold‐Multimer framework appear sufficient to construct some antibody–antigen complex structures with high accuracy, and through further training and optimization, success can potentially be improved for such complexes.

Although protein–protein interfaces were not used for training of AlphaFold v.2.0, it is possible that individual chain structures from BM5.5 complex structures were part of the AlphaFold training set, which could influence the predicted conformations of the subunits and indirectly influence the complex structure models. Benchmarking of this AlphaFold model with recently released complex structures that have no related complexes released prior to the AlphaFold training date addresses this concern, and results with such a set were reported by Evans et al. in the AlphaFold‐Multimer study,^36^ as well as this study. As most complexes in our BM 5.5 set are classified as rigid‐body (64%), with minimal conformational change between unbound and bound structure, the knowledge and possible use of the bound conformation, if used for training of the AlphaFold model, may have little effect or bias versus use of the unbound or accurately modeled unbound structure for that large subset of cases. Benchmarking of traditional docking methods with unbound‐bound cases (with one input protein taken from the bound complex structure rather than an unbound structure) could better reflect the use of knowledge of at least one bound component.

While this manuscript was under review, a separate study^57^ was published that also reported benchmarking of AlphaFold with a set of protein–protein complexes. While the authors used a distinct test set of 216 protein complexes from the Dockground protein docking benchmark,^21^ and employed a different MSA‐generation method, they reported a 63% success rate for acceptable or higher model quality, which is similar to our observed 51% success for models of that quality from AlphaFold. Furthermore, as in our study, the authors found that larger interface sizes were associated with improved AlphaFold success, and that interface residue‐based pLDDT scores were useful in model selection. However, Bryant et al. noted that size of paired MSAs resulted in improved AlphaFold success, as well as a possible greater dependence of protein source on AlphaFold success; those differences with our observations may be in part due to their use of their own optimized paired MSAs as AlphaFold input,^57^ while we obtained paired MSAs from ColabFold.^29^

AlphaFold's end‐to‐end modeling approach represents a major advance and performance improvement over traditional protein–protein docking methods, serving as a proof of concept and a possible framework for optimization to accurately model most or all protein–protein interactions. While optimization of AlphaFold for protein complexes (AlphaFold‐Multimer) was recently reported and released by the DeepMind team^36^ and was tested in this study, another team showed that combination of AlphaFold with a previously developed protein docking method was able to achieve an improvement in docking success,^49^ and others have shown the effects of optimized MSAs in AlphaFold complex modeling performance.^57^ Notably, a recent study used a combination of AlphaFold and RoseTTAFold to model structures of a large set of eukaryotic protein complexes.^58^ Prospective developments that build upon and optimize the AlphaFold framework, or utilize other geometric deep learning methods, can bring the field closer to solving the longstanding challenge of predictive protein docking.

## 4 MATERIALS AND METHODS

### 4.1 Protein–protein complex benchmark and additional antibody–antigen test cases

A test set of heterodimeric protein complexes was obtained from Protein–Protein Docking Benchmark 5.5 (BM5.5).^20^, ^23^ BM5.5 is a set of structures of nonredundant transient protein–protein complexes from the PDB,^37^ assembled for testing of predictive protein–protein complex modeling algorithms. By filtering for heterodimeric protein–protein complexes in BM5.5, we obtained a total of 152 cases, consisting of 12 antibody–antigen complexes, 72 enzyme‐containing complexes and 68 other types of protein complexes. Docking difficulty classifications for test cases were obtained from the BM5.5 site (https://zlab.umassmed.edu/benchmark/), and are based on the extent of binding conformational changes for each complex.^20^ Annotations of protein source organisms were obtained from the PDB, and confirmed by manual inspection. BSA for each complex interface was obtained from the BM5.5 site.

Twenty additional antibody–antigen modeling test cases (Table S4) were selected from antibody–antigen complex structures in the SAbDab database,^59^ screened by resolution (< 3.25 Å) and nonredundancy with any BM5.5 test cases by antibody chain sequences (< 90% antibody variable domain sequence identity) or antigen chain sequence (no hit to antigen chains using default parameters) using the “blastp” executable from the BLAST + suite.^60^ VLR‐antigen complex structure test cases (Table S5) were identified from the PDB and inspected manually for nonredundancy. Recently released antibody–antigen docking benchmark cases obtained from a preliminary update of BM5.5 (Table S6) and antibody–antigen complex structures identified from the SAbDab database (Table S7), filtered to remove complexes redundant with any complex structures that were released in the PDB before May 2018, were assembled for AlphaFold‐Multimer testing. As an additional nonredundancy check for the latter set of cases, we removed any antibody–antigen complexes with antigen BLAST hits (E‐value cutoff 5, and ≥ 40% identity) to antibody–antigen complex structures from pre‐May 2018, along with similar docking orientation (< 5 Å RMSD for heavy chain variable domain orientation after superposition of antigens using FAST structural alignment^61^) and > 70% sequence identity for heavy chain variable domain, light chain variable domain, or combined CDR sequences. For modeling efficiency, recently released and additional (non‐BM5.5) antibody structures were modeled with variable domains only.

TCR‐pMHC complex structures for AlphaFold‐Multimer benchmarking were identified from Class I TCR‐pMHC complex structures in the TCR3d database,^60^ and were originally obtained from the PDB. TCR‐pMHC complex structures were selected from structures released in the PDB after April 2018, with no redundancy (< 90% TCR variable domain sequence identity, in addition to < 95% sequence identity to any individual TCR *α* or *β* variable domain) with any Class I TCR‐pMHC complex structures released before May 2018, and no redundancy with any of the complex structures in the benchmark set. Complexes with noncanonical or modified amino acids in peptides were excluded, and a resolution cutoff of 3.25 Å was applied (in accordance with the other benchmarks in this study), except for the 7RM4 TCR‐pMHC complex structure which was retained due to its resolution being close to the cutoff (3.33 Å). TCR *α*, TCR *β*, peptide, and MHC chains were input as separate sequences to AlphaFold‐Multimer. For efficiency, only TCR variable domain sequences, and peptide‐binding domains of MHCs (*α*1 and *α*2 domains), were used for modeling.

### 4.2 Complex modeling with AlphaFold

AlphaFold was downloaded from Github (https://github.com/deepmind/alphafold) and installed on a local computing cluster. Sequences of protein chains for the protein–protein complexes were obtained from the PDB “seqres” file and used as input for each complex modeling job. Raw MSAs were prepared for each chain with the downloaded published AlphaFold pipeline,^24^ querying the full databases (UniRef90 version 2020_01, MGnify version 2018_12, Uniclust30 version 2018_08 and BFD). The resulting raw MSAs of the interacting chains were subsequently combined to form the unpaired MSA inputs for complex structure prediction. To generate MSA lines of the same length, gaps equal to the length of the interacting chain were added before or after each sequence. To avoid implicitly biasing the complex structure predictions with knowledge of individual bound protein chain conformations, the use of structural templates was disabled in this study.

To introduce chain breaks, a residue index shift of 200 was added to the junction of interacting chains, as recently implemented in ColabFold.^29^ Following the published AlphaFold pipeline, AlphaFold generated five models for each complex, which were ranked in this study by pTM score (which is a measure of predicted structure accuracy generated by AlphaFold). After structural predictions were generated, model relaxation by the Amber program,^63^ which as reported by Jumper et al.^24^ was used to ameliorate minor structural defects without impacting accuracy, was replaced by the constrained FastRelax protocol in the Rosetta program,^46^ as detailed below. To test the impact of varying the ensembling (*N*_ensemble_) and recycling (*N*_cycle_) parameters on complex modeling accuracy, we increased *N*_ensemble_ or *N*_cycle_ by modifying those parameters in the AlphaFold Python code, while keeping the input MSAs and sequences/features the same.

Three out of the 152 test cases failed to complete in the AlphaFold pipeline due to GPU memory limits during structure prediction, or errors during feature preparation: 1ZM4, 2OZA, and 1B6C. AlphaFold structure prediction runs were performed on an NVIDIA Titan RTX or NVIDIA Quadro 6,000 GPU.

### 4.3 Complex modeling with ColabFold and RoseTTAFold

Protein–protein complex predictions were generated in ColabFold^29^ using its “advanced” interface (https://colab.research.google.com/github/sokrypton/ColabFold/blob/main/beta/AlphaFold_advanced.ipynb). Input protein sequences were identical to those used for AlphaFold modeling. The MMseqs2 method^62^ was selected on ColabFold to generate the MSAs, and Amber relaxation of models was disabled. The unpaired MSA predictions were generated between August 20 and August 24, 2021.

ColabFold enables users to pair alignments for different protein sequences based on UniProt accession numbers; this is a selectable option on the ColabFold interface.^29^ Since the protocol is designed to pair prokaryotic protein sequences, MSA pairing was only performed on a subset of cases where both protein chains of the heterodimer complex come from the same prokaryotic organism. Prefiltering of MSAs was enabled prior to pairing, with the minimum coverage with query of 50% and minimum sequence identity with query of 20%. Structural predictions based on paired MSAs were generated on September 4, 2021. The resulting paired MSAs were also used as input to RoseTTAFold^28^ on the Robetta web server (https://robetta.bakerlab.org/submit.php) to generate complex models.

All models generated with AlphaFold, ColabFold and RoseTTAFold are made available to the public at: https://piercelab.ibbr.umd.edu/af_complex_models.html.

### 4.4 Complex modeling with AlphaFold‐Multimer

AlphaFold‐Multimer^36^ modeling was performed with AlphaFold downloaded from https://github.com/deepmind/alphafold on November 2, 2021, and local ColabFold downloaded from https://github.com/sokrypton/ColabFold on January 12, 2021. Input MSA features were generated by either the AlphaFold‐Multimer pipeline described in Evans et al.,^36^ or by local ColabFold^29^ using the “MMseqs2 (Uniref + Environmental)” MSA mode. By default, the MSAs constructed contain both unpaired (per‐chain) and paired sequences. To generate AlphaFold‐Multimer predictions using alternative MSA pairing modes (“unpaired only,” “paired only,” or “no MSA”), local ColabFold was used. Specifically, MSA pair mode was set to “Paired” to generate “paired only” predictions. The MSA pair mode was set to “unpaired + paired” to generate “unpaired only” predictions, and “paired” to generate “no MSA” predictions, after modifying the “get_msa_and_templates” function in “batch.py” of local ColabFold, so the list variable “paired_a3m_lines” contains only the query sequences, instead of paired sequences generated by MMSeqs2. While “no MSA” (a.k.a. “single sequence”) and “unpaired” options are available in the ColabFold Google Colab interface, we found the above modification necessary in the version of the ColabFold code that we downloaded at the time. To avoid implicitly biasing the structural predictions with knowledge of known conformations, a template release date cutoff of April 30, 2018 was applied when the use of templates was enabled.

### 4.5 Docking model generation with ZDOCK

To enable comparison against a rigid‐body docking algorithm, we generated docking models using ZDOCK version 3.0.2.^33^ Unbound protein structures from BM5.5, with HETATMs removed, were used as inputs to ZDOCK. Dense rotational sampling was used, generating 54,000 predictions per complex. The integration of residue‐ and atom‐based potentials for docking (IRAD)^39^ scoring function was used to rank the ZDOCK output models.

### 4.6 Docking model accuracy assessment

Docking models were assessed using the CAPRI criteria^22^ using custom scripts. Based on the structural similarity between docking models and native structures, docking models were classified into four accuracy classes: “high,” “medium,” “acceptable” and “incorrect”. Such structural similarity is assessed by a combination of interface RMSD (I‐RMSD), ligand RMSD (L‐RMSD), and *f*_nat_. Backbone atoms were used in the I‐RMSD and L‐RMSD calculations.

### 4.7 Interface pLDDT and interface PAE calculation

To calculate the interface pLDDT, we averaged the per‐residue pLDDT of interface residues. Interface residues are defined as residues with atomic contacts across the interface within the specified distance cutoff. Interface PAE was calculated by averaging the PAE of cross‐interface residue pairs with atomic contacts within a given distance cutoff. The interface distance cutoffs tested range from 4 to 10 Å. An interface pLDDT score of 0 and an interface PAE score of 35 was assigned to models without any interface contacts within the distance cutoff.

### 4.8 Structure relaxation using Rosetta

To resolve possible unfavorable geometries or clashes in experimentally determined complex structures and AlphaFold models, the Rosetta FastRelax protocol^64^ was applied to the predicted structures prior to scoring of the models using interface analysis protocols (IRAD, ZRANK2, and Rosetta). Parameter flags used in FastRelax (“relax” executable in Rosetta 3.12^44^) are:

‐relax:constrain_relax_to_start_coords

‐relax:coord_constrain_sidechains

‐relax:ramp_constraints false

‐ex1

‐ex2

‐use_input_sc

‐no_optH false

‐flip_HNQ

‐nstruct 1

### 4.9 Complex and docking model scoring with IRAD, ZRANK2, and Rosetta InterfaceAnalyzer

Post‐relax complex structures were used as inputs to obtain IRAD,^39^ ZRANK2,^15^ and Rosetta^46^ InterfaceAnalyzer protocol scores. IRAD and ZRANK2 scores were obtained from the downloaded “irad” executable program. InterfaceAnalyzer scores were obtained using the. “InterfaceAnalyzer” executable in Rosetta v. 3.12, with default parameters; the InterfaceAnalyzer protocol computes and outputs interface energetic scores using the Rosetta REF15 function,^67^ along with REF15 component terms and other interface structure metrics.

### 4.10 Number of effective sequences

The *N*_eff_ is used as a measure of the MSA depth. *N*_eff_ score is defined as the number of clusters after the raw MSA inputs were clustered at the 62% sequence identity using CD‐HIT^66^ with the word length of 4, as used previously.^67^

### 4.11 TM‐score calculations

TM‐scores were calculated using TM‐score executable^34^ by comparing the structural similarity between experimentally determined structures and AlphaFold models. Residues that were unresolved in experimentally determined structures were removed from AlphaFold models before the calculation of TM‐scores.

### 4.12 Figures, statistical analysis, and AUC calculations

Figures of structures were generated using PyMOL version 2.4 (Schrodinger, Inc.). Box plots, line plots and bar plots were generated with the ggplot2 package^68^ in R (r-project.org), and heatmaps were generated with the ComplexHeatmap package^69^ in R. Pearson correlations and their *p* values were calculated with ggpubr package in R. Wilcoxon rank‐sum test was performed using ggsignif package in R. Binary and multi‐class ROC curves with AUC values were calculated with the “pROC” and “multiROC” packages, respectively, in R. 95% CI values for binary ROC AUC values were calculated using the “ci.auc” function in pROC, with 2000 stratified boostrap replicates, and multi‐class ROC AUC confidence interval values were calculated using the “boot” R package^70^ with the adjusted bootstrap percentile method. Calculations of possible score thresholds for model selection were performed using the “cutpointr” package,^71^ with maximization of the sum of the sensitivity and specificity, based on discrimination of incorrect versus medium/high‐CAPRI accuracy models.

## AUTHOR CONTRIBUTIONS

**Rui Yin:** Conceptualization; data curation; formal analysis; investigation; writing – original draft; writing – review and editing. **Brandon Y Feng:** Writing – original draft; writing – review and editing. **Amitabh Varshney:** Writing – review and editing. **Brian G Pierce:** Conceptualization; funding acquisition; supervision; writing – original draft; writing – review and editing.

## CONFLICT OF INTEREST

The authors declare no competing interests.

## Supporting information


**Appendix S1** Supporting Information.
