# Supplementary material for: Benchmarking AlphaFold for protein complex modeling reveals accuracy determinants
Source: Protein Sci. 2022 Jul 13;31(8):e4379. doi: 10.1002/pro.4379 (PMC9278006; doi:10.1002/pro.4379)
Supplement: Supplementary file 1 — Appendix S1 Supporting Information. [file PRO-31-e4379-s001.pdf]

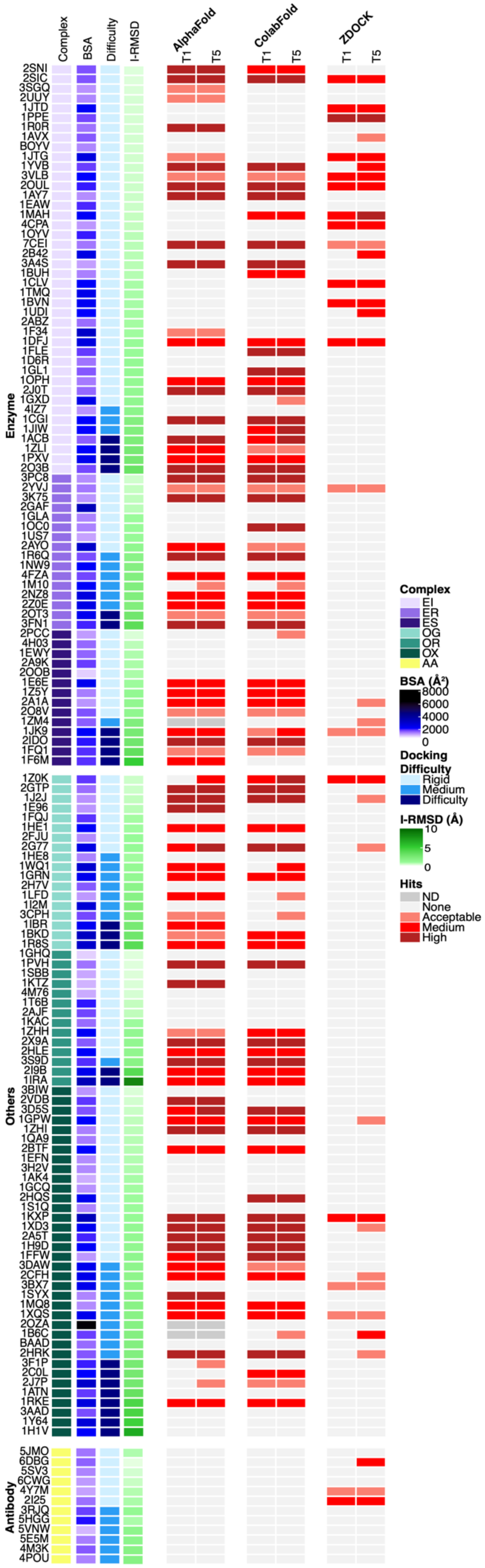

Figure S1. Predictive modeling success of AlphaFold, ColabFold, and ZDOCK. Cases are grouped and sorted by complex type. For reference, complex category, buried surface area (BSA, Å²), docking difficulty based on binding conformational changes, and binding interface root mean square distance (I-RMSD) are indicated on the left. Complex category: Enzyme-inhibitor (EI), enzyme complex with a regulatory or accessory chain (ER), enzyme-substrate (ES), others, G-protein containing (OG), others, receptor-containing (OR); others, miscellaneous (OX), antibody-antigen (AA). Success for top 1 and top 5 (T1, T5) ranked predictions is shown, colored by CAPRI model accuracy as indicated in the key on right (Hits). Three cases failed to complete in AlphaFold and are shown as dark gray cells ("ND" under Hits).

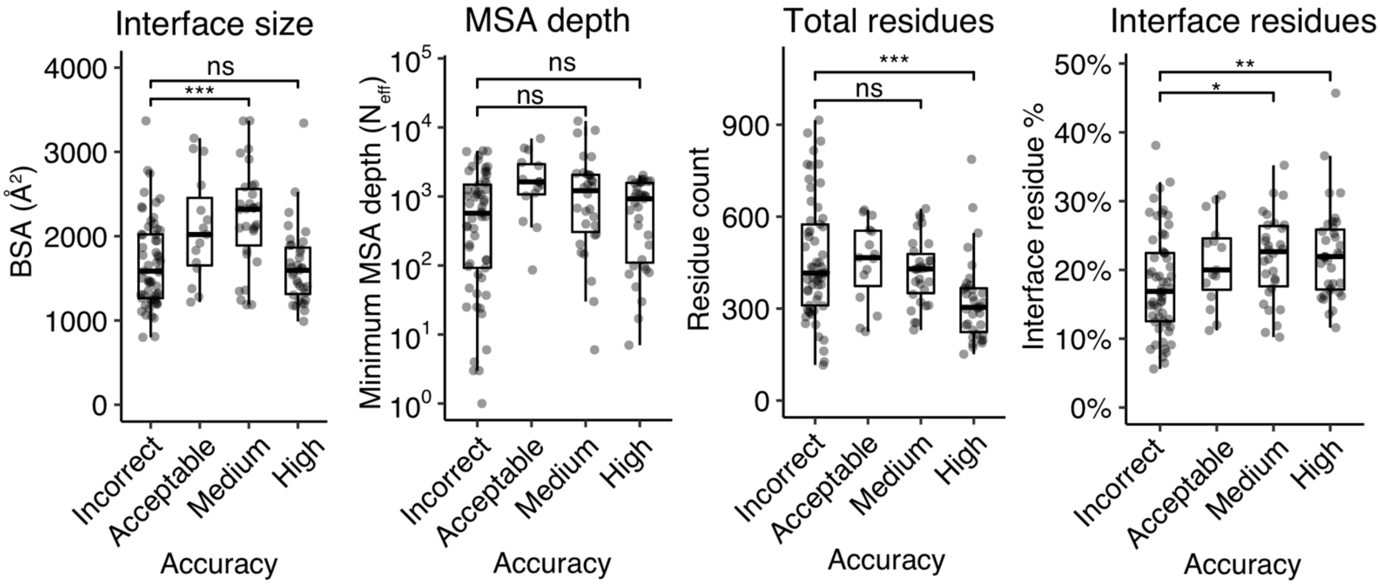

Figure S2. Assessment of features associated with AlphaFold success or lack of success, with antibody complexes excluded. Protein complex and MSA feature values were computed for all cases except for antibody-antigen complexes, shown according to AlphaFold success (best AlphaFold model accuracy in the five models for that case). Features shown are interface buried surface area (BSA), MSA depth (N_{eff}) for the ligand or receptor (minimum value of the two), total number of residues, and percent of total residues in protein-protein interface. Statistical significance values (Wilcoxon rank-sum test) were calculated between feature values for sets of cases with Incorrect vs. Medium and Incorrect vs. High CAPRI accuracy, as noted at top (ns: $p > 0.05$, *: $p \leq 0.05$, **: $p \leq 0.01$, ***: $p \leq 0.001$).

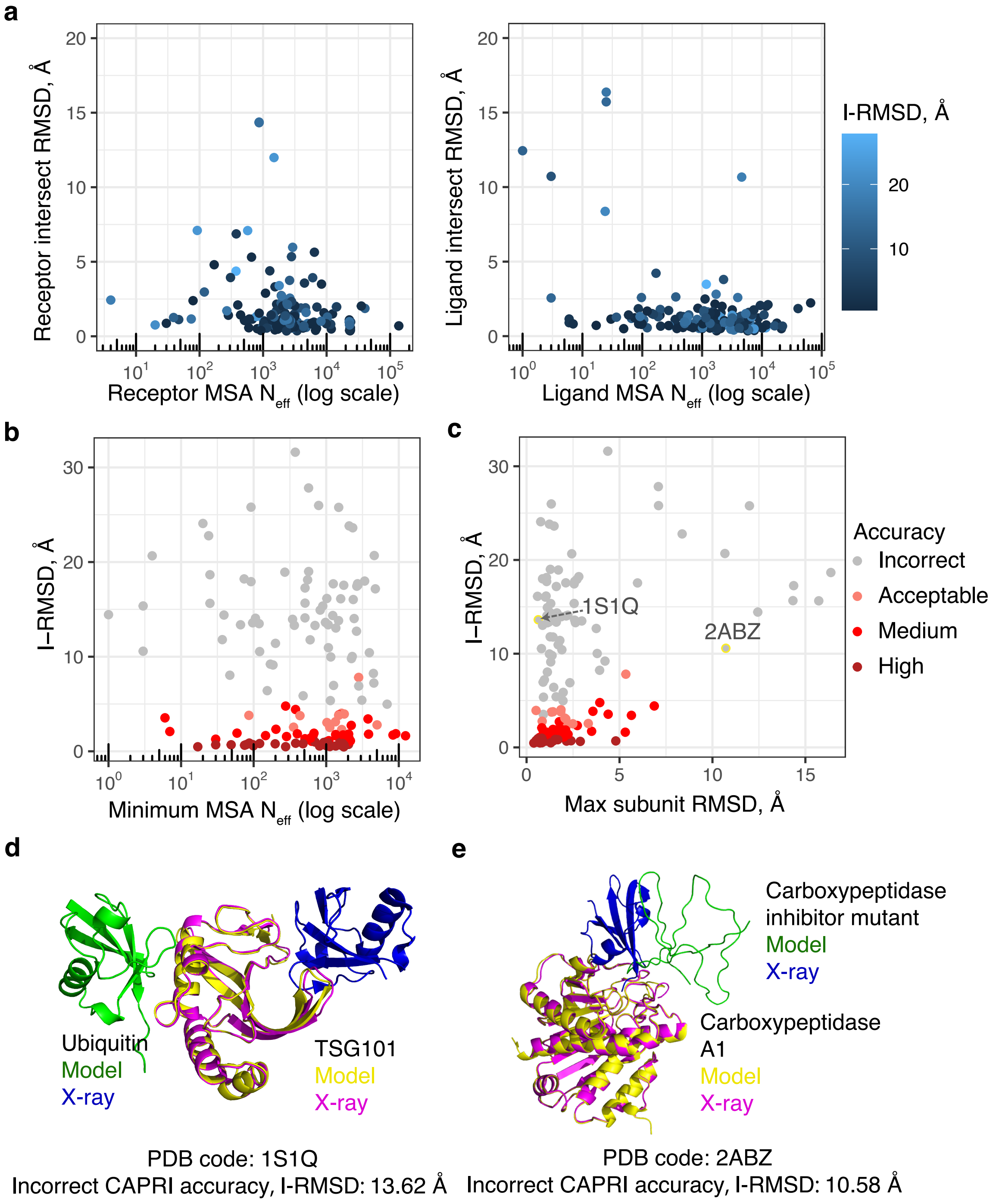

Figure S3. Comparison of MSA depth, subunit accuracy, and AlphaFold model quality. (a) Scatter plots depicting the relationship between ligand or receptor intersect RMSD and MSA depth (log scale; measured by N_{eff}, see Materials and Methods for details). Each point represents the top pTM model from each of the 149 cases modeled by AlphaFold. Points are colored by I-RMSD of the docking model. Scatter plot depicting the relationship between the docking model I-RMSD and (b) minimum ligand and receptor MSA depth (log scale, measured by N_{eff}), or (c) Maximum subunit (ligand or receptor) RMSD from X-ray structure. Points are colored by CAPRI accuracy and represent the top-ranked model (by pTM score) from each of the 149 cases modeled by AlphaFold. (d) Native and top-ranked AlphaFold model (pTM = 0.89) for PDB 1S1Q, superposed by TSG101. (e) Native and top-ranked AlphaFold model (pTM = 0.81) for PDB 2ABZ, superposed by Carboxypeptidase A1. Model and the X-ray structure chains are colored separately as indicated. Unresolved regions modeled by AlphaFold were omitted from the figures.

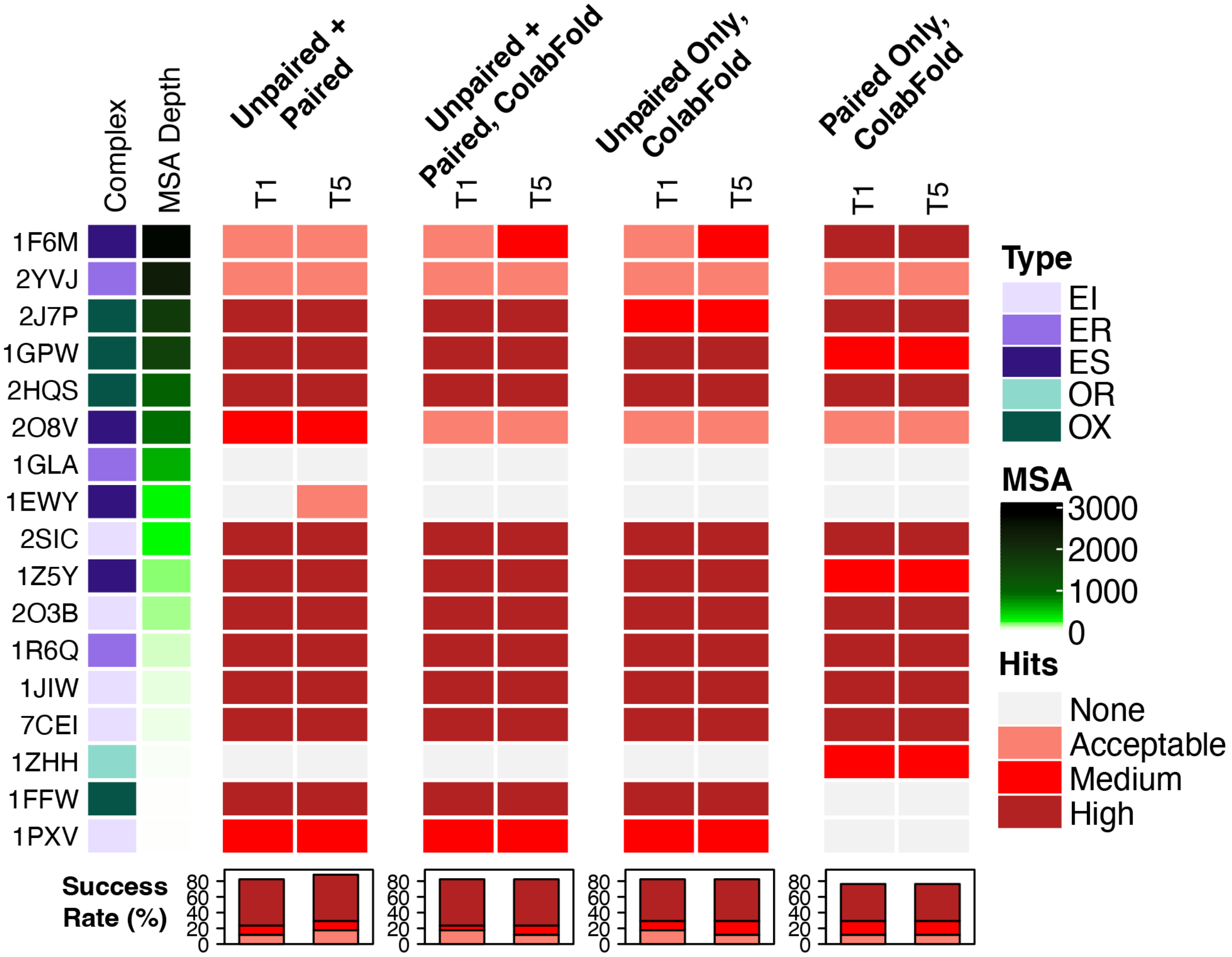

Figure S4. Testing the impact of MSA modes on AlphaFold-Multimer accuracy. Models were generated with AlphaFold-Multimer without templates on a set of 17 cases in from BM5.5 possessing chains from same prokaryotic species. Modeling was performed locally using AlphaFold-Multimer from the DeepMind Github downloaded protocol, or AlphaFold-Multimer in ColabFold, with the latter noted in column headers. All models were assessed for CAPRI accuracy, and model quality of top 1 and top 5 predictions (ranked by AlphaFold-Multimer score, 0.8*ipTM + 0.2*pTM) is shown and colored by CAPRI model accuracy as indicated in the key on right (Hits). Cases are sorted by the depth of the paired MSA (N_{eff}), generated by the AlphaFold-Multimer protocol in ColabFold, from the largest to the smallest values.

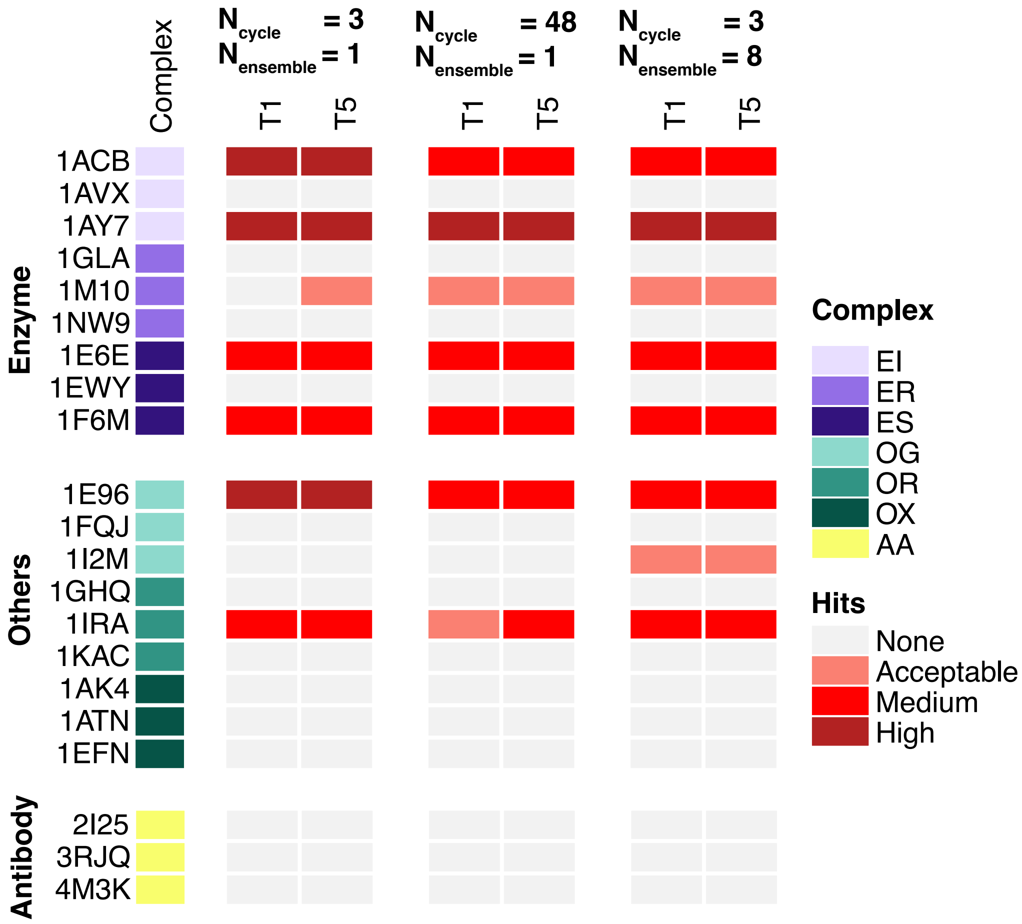

Figure S5. Testing the impact of alternative parameters on AlphaFold accuracy. Models were generated with AlphaFold for 21 test cases, representing three cases from each of the 7 complex categories, chosen by random. By default, N_{cycle} is set to 3 and $N_{ensemble}$ is set to 1. To test the impact of using alternative parameters, N_{cycle} and $N_{ensemble}$ were respectively increased to 48 and 8, while all other components of AlphaFold pipeline were kept constant. All models were assessed for near-native predictions using CAPRI criteria for High, Medium and Acceptable accuracy in the top-ranked (T1) and top 5 (T5) models. Complex category: Enzyme-inhibitor (EI), enzyme complex with a regulatory or accessory chain (ER), enzyme-substrate (ES), others, G-protein containing (OG), others, receptor-containing (OR); others, miscellaneous (OX), antibody-antigen (AA).

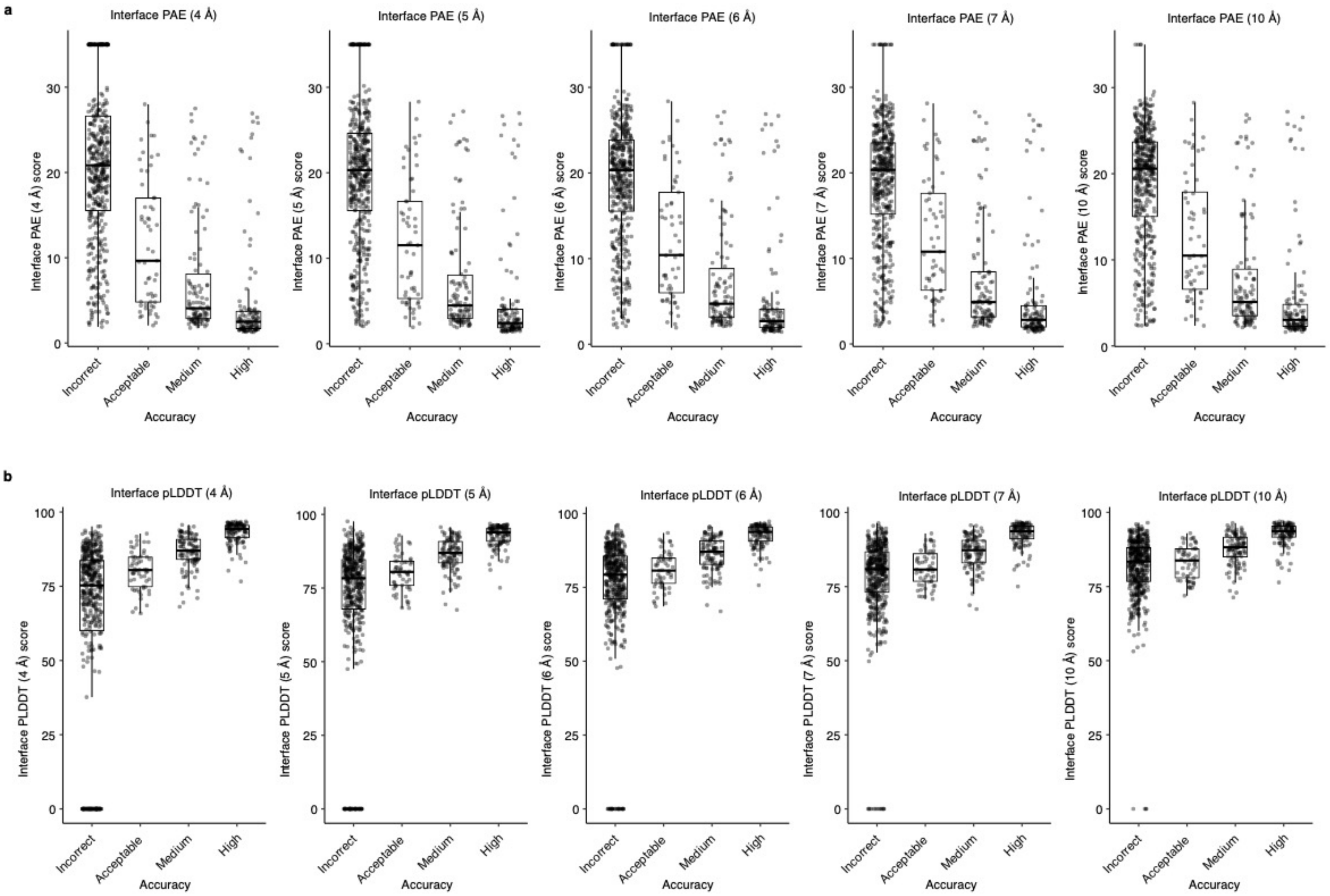

Figure S6. Alternative formulation of interface accuracy metrics calculated from AlphaFold predictions (PAE, pLDDT) (a) Interface PAE within a distance cutoff of 4 Å, 5 Å, 6 Å, 7 Å and 10 Å grouped by docking model accuracy. (b) Interface pLDDT within a distance cutoff of 4 Å, 5 Å, 6 Å, 7 Å and 10 Å, grouped by docking model accuracy. An interface pLDDT score of 0 and an interface PAE score of 35 are assigned to models without interface contacts within the distance cutoff specified. See also Table S2 for AUC values for interface pLDDT and interface PAE as classifiers for docking model accuracy.

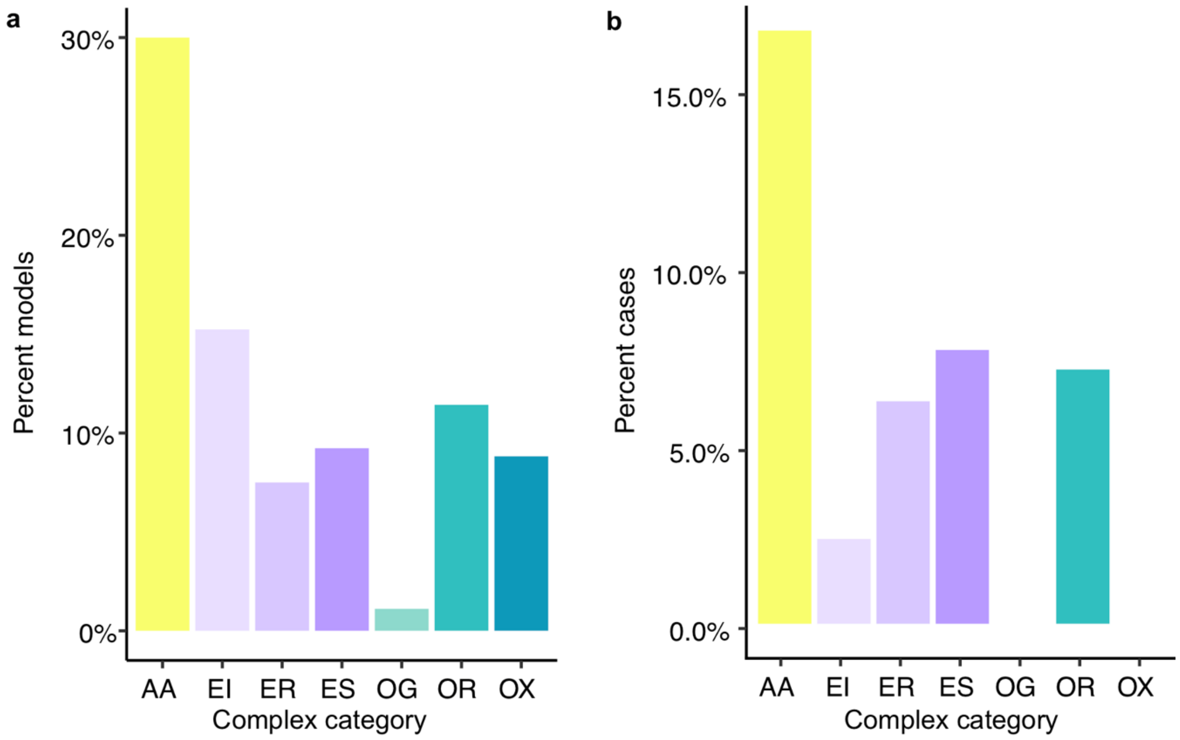

Figure S7. **Distribution of AlphaFold models without inter-chain atomic contacts by complex category.** (a) Percentage of AlphaFold models without inter-chain atomic contacts within 4 Å distance cutoff per complex category. (b) Percentage of cases in which the top-ranked model (ranked by pTM) predicted by AlphaFold has no inter-chain atomic contacts within 4 Å distance cutoff in each complex category. Complex category: Antibody-antigen (AA), enzyme-inhibitor (EI), enzyme complex with a regulatory or accessory chain (ER), enzyme-substrate (ES), others, G-protein containing (OG), others, receptor-containing (OR); others, miscellaneous (OX).

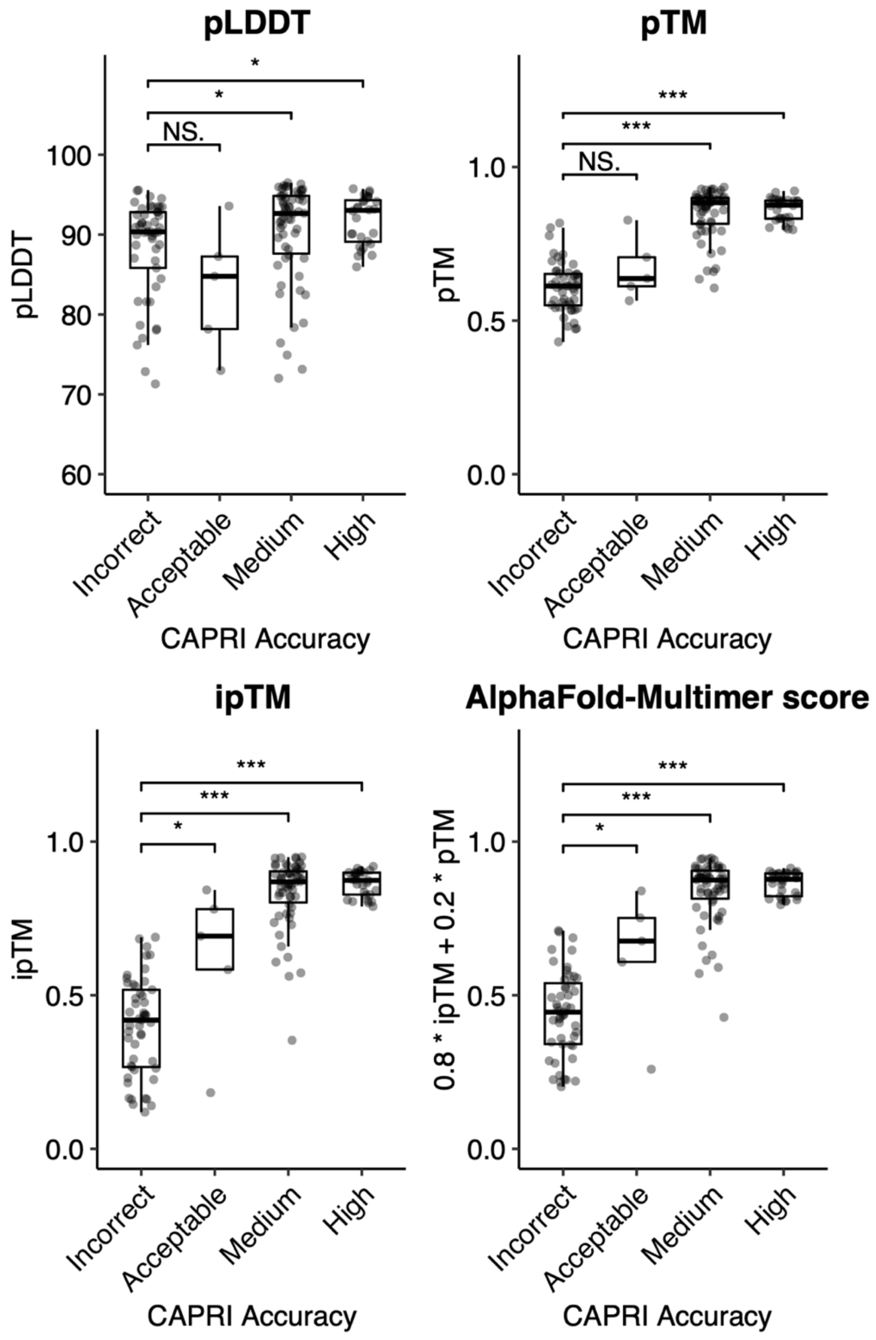

Figure S8. Discrimination of AlphaFold-Multimer model accuracies with pLDDT, pTM, ipTM, and AlphaFold-Multimer model scores. A benchmark set of recently released complex structures (from Table 2) was modeled with AlphaFold-Multimer, and all five models from AlphaFold-Multimer per test case were assessed for accuracy using CAPRI criteria. Accuracy-binned models are shown in comparison with pLDDT, pTM, ipTM, and AlphaFold-Multimer (0.8*ipTM + 0.2*pTM) scores. Statistical significance values (Wilcoxon rank-sum test) are shown for group comparisons (NS: $p > 0.05$, *: $p \leq 0.05$, ***: $p \leq 0.001$).

Table S1. Heterodimeric protein-protein complexes from BM5.5 tested in this study.

PDB code	Receptor	Ligand	Complex Category[a]	BSA (Å^2)[b]	Protein source[c]
1ACB	Chymotrypsin	Eglin C	EI	1544	ME
1AK4	Cyclophilin	HIV capsid	OX	1029	MM
1ATN	Actin	Dnase I	OX	1774	ME
1AVX	Porcine trypsin	Soybean trypsin inhibitor	EI	1585	ME
1AY7	RNase Sa	Barstar	EI	1237	MB
1B6C	FKBP binding protein	TGFbeta receptor	OX	1752	SE
1BKD	Ras GTPase	Son of sevenless	OG	3163	SE
1BUH	CDK2 kinase	Ckshs1	EI	1324	SE
1BVN	Alpha-amylase	Tendamistat	EI	2222	MM
1CGI	Bovine chymotrypsinogen	PSTI	EI	2053	ME
1CLV	Alpha-amylase	Alpha-amylase inhibitor	EI	2087	ME
1D6R	Bovine trypsin	Bowman-Birk inhibitor	EI	1408	ME
1DFJ	Ribonuclease A	Rnase inhibitor	EI	2582	ME
1E6E	Adrenoxin reductase	Adrenoxin	ES	2315	SE
1E96	Rac GTPase	p67 Phox	OG	1179	SE
1EAW	Matriptase	BPTI	EI	1866	ME
1EFN	HIV-1-NEF protein	SH3 domain	OX	1254	MM
1EWY	Ferredoxin reductase	Ferredoxin	ES	1502	SB
1F34	Porcine pepsin	Ascaris inhibitor 3	EI	3038	ME
1F6M	Thioredoxin reductse	Thioredoxin 1	ES	1821	SB
1FFW	Chemotaxis protein CheY	Chemotaxis protein CheA	OX	1166	SB
1FLE	Elastase	Elafin	EI	1763	ME
1FQ1	CDK2 kinase	CDK inhibitor 3	ES	1832	SE
1FQJ	Gt-alpha	RGS9	OG	1806	ME
1GCQ	GRB2 C-ter SH3 domain	Vav N-ter SH3 domain	OX	1208	ME
1GHQ	Complement C3	Epstein-Barr virus receptor CR2	OR	800	SE
1GL1	Alpha-chymotrypsin	Protease inhibitor LCMI II	EI	1591	ME
1GLA	Glycerol Kinase	Glucose specific phosphocarrier	ER	1304	SB
1GPW	HISF protein	Amidotransferase HISH	OX	2097	SB
1GRN	CDC42 GTPase	CDC42 GAP	OG	2332	SE
1GXD	proMMP2 type IV collagenase	Metalloproteinase inhibitor 2	EI	2445	SE
1H1V	Actin	Gelsolin	OX	2071	ME
1H9D	Runx1 domain of CBFAlpha1	Dimerisation domain of CBF-Beta	OX	2121	SE
1HE1	Rac GTPase	Pseudomonas toxin GAP dom.	OG	2113	MM
1HE8	Ras GTPase	PIP3 kinase	OG	1305	SE
1I2M	Ran GTPase	RCC1	OG	2779	SE
1IBR	Ran GTPase	Importin beta	OG	2270	SE
1IRA	Interleukin-1 receptor	Interleukin-1 receptor antagonist protein	OR	3367	SE
1J2J	Arf1 GTPase	GAT domain of GGA1	OG	1209	ME
1JIW	Alkaline metalloproteinase	Proteinase inhibitor	EI	1997	SB
1JK9	CCS metallochaperone	SOD1 superoxide dismutase	ES	2130	SE
1JTD	BLIP-II	TEM-1 beta-lactamase	EI	2180	MB
1JTG	Beta-lactamase inhibitor protein	Beta-lactamase TEM-1	EI	2600	MB
1KAC	Adenovirus fiber knob protein	Adenovirus receptor	OR	1456	MM
1KTZ	TGF-Beta	TGF-Beta receptor	OR	989	SE
1KXP	Actin	Vitamin D binding protein	OX	3341	ME
1LFD	Ras	RalGDS Ras-interacting domain	OG	1167	ME
1M10	Von willebrand factor dom. A1	Glycoprotein IB-Alpha	ER	2097	SE
1MAH	Acetylcholinesterase	Fasciculin	EI	2145	ME
1MQ8	ICAM-1 domain 1-2	Integrin Alpha-L I domain	OX	1253	SE
1NW9	Capase-9	BIR3-XIAP	ER	2112	SE
1OC0	Plasminogen activator inhibitor-1	Vitronectin Somatomedin B domain	ER	1313	SE
1OPH	Alpha-1-antitrypsin	Trypsinogen	EI	1360	ME
1OYV	Subtilisin Carlsberg	Two-headed tomato inhibitor-II	EI	1930	MM
1PPE	Bovine trypsin	CMTI-1 squash inhibitor	EI	1688	ME
1PVH	IL6 receptor Beta chain D2-D3 domains	Leukemia inhibitory factor	OR	1403	SE

1PXV	Cystein protease	Cystein protease inhibitor	EI	2336	SB
1QA9	CD2	CD58	OX	1353	SE
1R0R	Subtilisin carlsberg	OMTKY	EI	1409	MM
1R6Q	Clp protease subunit ClpA	Clp protease adaptor protein ClpS	ER	1651	SB
1R8S	Arf1 GTPase	Sec 7 domain	OG	2986	ME
1RKE	Vinculin head	Vinculin tail	OX	2614	SE
1S1Q	UEV domain	Ubiquitin	OX	1288	SE
1SBB	T-cell receptor Beta	Staphylococcus enterotoxin B	OR	1064	MM
1SYX	Spliceosomal U5 15 kDa protein	CD2 receptor binding protein 2 C-ter fragment	OX	1293	SE
1T6B	Anthrax protective antigen	Anthrax toxin receptor	OR	1948	MM
1TMQ	alpha-amylase	RAGI inhibitor	EI	2401	ME
1UDI	Uracyl-DNA glycosylase	Glycosylase inhibitor	EI	2022	Viral
1US7	Heat shock protein 82 N-ter domain	HSP 90 co-chaperone CDC37 C-ter domain	ER	1095	ME
1WQ1	Ras GTPase	Ras GAP	OG	2913	SE
1XD3	UCH-L3	Ubiquitin	OX	2281	SE
1XQS	HspBP1	Hsp70 ATPase domain	OX	2350	SE
1Y64	Actin	BNI1 protein	OX	2745	ME
1YVB	Falcipain 2	Cystatin	EI	1743	ME
1Z0K	Rab4A GTPase	RAB4 binding domain of Rabenosyn	OG	1787	SE
1Z5Y	N-term of DsbD	E.coli CCMG protein	ES	1346	SB
1ZHH	Autoinducer 2-binding periplasmic protein LuxP	Autoinducer 2 sensor kinase/phosphatase LuxQ	OR	2189	SB
1ZHI	BAH domain of Orc1	Sir Orc-interaction domain	OX	1322	SE
1ZLI	Carboxypeptidase B	Tick carboxypeptidase inhibitor	EI	2084	ME
1ZM4	Elongation factor 2	Diphtheria toxin A catalytic domain	ES	1554	MM
2A1A	Eukayotic translation initiation factor 2-alpha kinase 2	eIF2 alpha subunit	ES	1186	ME
2A5T	NMDA receptor R1-4A subunit ligand-binding core	NMDA receptor R2A subunit ligand-binding core	OX	1892	ME
2A9K	Ras-related protein Ral-A	Mono-ADP-ribosyltransferase C3	ES	1751	MM
2ABZ	Carboxypeptidase A1	Leech carboxypeptidase inhibitor	EI	1443	ME
2AJF	ACE2	SARS spike protein receptor binding domain	OR	1704	MM
2AYO	Ubiquitin carboxyl-terminal hydrolase 14	Ubiquitin	ER	3027	SE
2B42	Xylanase	Xylanase inhibitor	EI	2520	MM
2BTF	Actin	Profilin	OX	2063	SE
2C0L	PTS1 and TRP region of PEX5	SCP2	OX	2013	SE
2CFH	BET3	TPC6	OX	2384	SE
2FJU	Phospholipase Beta 2	Rac GTPase	OG	1245	SE
2G77	GTPase-activating protein GYP1	Ras-related protein Rab-33B	OG	2524	ME
2GAF	Poly(A) polymerase VP55	Vaccinia protein VP39	ER	3368	Viral
2GTP	Alpha-1 subunit Guanine nucleotide-binding protein G(I), alpha-1 subunit	RGS1	OG	1442	SE
2H7V	Rac GTPase	YpkA	OG	1574	MM
2HLE	Ephrin B4 receptor	Ephrin B2 ectodomain	OR	2116	SE
2HQS	TolB	Pal	OX	2333	SB
2HRK	Glutamyl-t-RNA synthetase	GU-4 nucleic binding protein	OX	1595	SE
2I25	Shark single domain antigen receptor	Lysozyme	AA	1425	ME
2I9B	Urokinase plasminogen activator surface receptor	Urokinase-type plasminogen activator	OR	2371	SE
2IDO	DNA polymerase III Œμ exonuclease domain	HOT protein (P1 phage)	ES	1953	MM
2J0T	MMP1 Intersitial collagenase	Metalloproteinase inhibitor 1	EI	1477	SE
2J7P	SRP GTPase Ffh	Cell division protein FtsY	OX	3008	SB
2NZ8	Rac GTPase	DH/PH domain of TRIO	ER	2599	SE
2O3B	NucA nuclease	NuiA nuclease inhibitor	EI	1675	SB
2O8V	PAPS reductase	Thioredoxin	ES	1619	SB
2OOB	Ubiquitin ligase	Ubiquitin	ES	808	ME
2OT3	Rab21 GTPase	Rabex-5 VPS9 domain	ER	2306	SE

2OUL	Falcipain 2	Chagasin	EI	1933	ME
2OZA	MAP kinase 14	MAP kinase-activated protein kinase 2	OX	6248	ME
2PCC	Cyt C peroxidase	Cytochrome C	ES	1141	SE
2SIC	Subtilisin	Streptomyces subtilisin inhibitor	EI	1617	MB
2SNI	Subtilisin	Chymotrypsin inhibitor 2	EI	1628	MM
2UUY	Trypsin	Tryptase inhibitor from tick	EI	1280	ME
2VDB	Serum albumin	Peptostreptococcal albumin-binding protein	OX	1798	MM
2X9A	TolA C-terminal domain	G3P TolA binding domain	OR	1571	MM
2YVJ	Ferredoxin reductase BPHA4	Biphenyl dioxygenase ferredoxin subunit	ER	1377	SB
2Z0E	Cysteine protease Atg4B	Microtubule-associated proteins 1A/1B light chain 3B	ER	2478	ME
3A4S	SUMO-conjugating enzyme UBC9	NFATC2-interacting protein SLD2 ubiquitin-like domain	EI	1116	ME
3AAD	Double bromodomain	Histone chaperone ASF1	OX	1654	SE
3BIW	Neuroligin-1	Neuroligin-1-beta	OX	1191	SE
3BX7	Lipocalin 2	CTLA-4 extracellular domain	OX	2349	SE
3CPH	Rab GDP-dissociation inhibitor	Ras-related protein Sec4	OG	1685	SE
3D5S	Complement C3d fragment	Fibrinogen-binding protein C-ter domain	OX	1620	MM
3DAW	Alpha actin	Twinfilin-1 C-terminal domain	OX	2323	ME
3F1P	HIF2 alpha C-terminal PAS domain	ARNT C-terminal PAS domain	OX	1919	SE
3FN1	UQ_con domain from NEDD8-conjugating enzyme UBE2F	NEDD8-activaating enzyme E1 catalytic subunit	ER	1897	SE
3H2V	Vinculin tail domain	Raver1 RRM1 domain	OX	1263	SE
3K75	DNA polymerase beta	Reduced XRCC1, N-terminal domain	ER	1195	ME
3PC8	DNA repair protein XRCC1	DNA ligase III-alpha BRCT domain	ER	1240	ME
3RJQ	A12	C1086 HIV gp120	AA	1734	MM
3S9D	IFNAR2	IFNa2	OR	1841	SE
3SGQ	Streptogrisin B	Ovomucoid inhibitor third domain	EI	1211	MM
3VLB	EDGP	Xyloglucan-specific endo-beta-1,4-glucanase A	EI	2020	ME
4CPA	Carboxypeptidase A	Potato carboxypeptidase inhibitor	EI	1175	ME
4FZA	MO25 alpha	Serine/threonine-protein kinase MST4	ER	1695	SE
4H03	Iota toxin component IA	Alpha actin	ES	1474	MM
4IZ7	Non-phosphorylated ERK	PEA-15 Death Effector Domain	EI	1202	ME
4M3K	cAb-H7S	B. licheniformis beta-lactamase	AA	1588	MM
4M76	C3D	Integrin alpha-M CD11B A-domain	OR	1046	SE
4POU	VHHmetal	bovine RNase A	AA	1313	ME
4Y7M	nb25	E coli TssM CTD	AA	1103	MM
5E5M	H11	mouse CTLA-4	AA	1341	ME
5HGG	Nb4	uPA	AA	1969	ME
5JMO	Nb14	Furin	AA	1394	ME
5SV3	A3C8	Ricin	AA	1294	ME
5VNW	Nb.b201	human serum albumin	AA	967	MM
6CWG	A9	Ricin	AA	1151	ME
6DBG	R303	Listeria monocytogenes internalin B	AA	1525	MM
7CEI	Colicin E7 nuclease	Im7 immunity protein	EI	1384	SB
BAAD	Double bromodomain	Histone chaperone ASF1	OX	1461	SE
BOYV	Subtilisin Carlsberg	Two-headed tomato inhibitor-II	EI	1280	MM

[a] Complex category: Antibody-antigen (AA), enzyme-inhibitor (EI), enzyme-substrate (ES), enzyme complex with a regulatory or accessory chain (ER), others, G-protein containing (OG), others, receptor-containing (OR); others, miscellaneous (OX).
[b] Interface buried surface areas.
[c] Based on the source organisms for receptor and ligand protein chains in the Protein Data Bank (PDB) [1], each case is classified as SE ("Single, Eukaryotic", denoting proteins from the same eukaryotic organism), SB ("Single, Bacterial", denoting proteins from the same bacterial organism), ME ("Multiple, Eukaryotic", denoting proteins from different eukaryotic organisms), MB ("Multiple, Bacterial", denoting proteins from different bacterial organisms), Viral (denoting proteins from viruses), or MM ("Multiple, Mix", denoting proteins from mixed origins).

Table S2. Analysis of geometric and energetic interface features associated with AlphaFold success or failure.

Interface Feature[a]	p-value Incorrect vs. Medium/High[b]	p-value Incorrect vs. High[b]
nres_tot	**0.02001**	**0.00022**
SASA_Tot	0.21257	**0.00253**
nres_percent	**0.00091**	**0.00412**
BSA_percent	**0.00038**	**0.00605**
per_residue_energy_int	0.53604	**0.04757**
hbond_E_fraction	0.19513	0.12584
sc_value	0.34752	0.18198
dG_cross	**0.00432**	0.22077
packstat	0.94851	0.34572
nres_int	0.07936	0.58908
ZRANK2	**0.00195**	0.66473
BSA	**0.00736**	0.84187
delta_unsatHbonds	0.19421	0.92855
hbonds_int	0.17700	0.99173

[a]Interface geometric or energetic feature, calculated using experimentally determined complex structures with the InterfaceAnalyzer protocol in Rosetta[2], with the exception of ZRANK2, SASA_Tot, BSA, BSA_percent, and nres_percent values. ZRANK2 scores were calculated with the ZRANK executable[3], SASA_Tot and BSA represent the total accessible surface area of the individual proteins and the buried interface surface area, calculated by naccess[4]. BSA_percent and nres_precent represent the proportion of total surface area or total residues in the interface, calculated using BSA and SASA_Tot (BSA_percent), or nres_tot and nres_int (nres_percent).
[b]P-values were calculated using Wilcoxon rank-sum test for sets of BM5.5 cases based on AlphaFold accuracy in the five models generated for that complex (all Incorrect, at least one model with Medium or High accuracy, at least one model with High accuracy). Significant p-values ($p < 0.05$) are shown in bold.

Table S3. Area under the ROC curve (AUC) value for protein quality classes as a function of interface scores calculated from AlphaFold predictions.

Score[a]	Binary classification		Multi-class classification
	Incorrect vs. High	Incorrect vs. Medium and High	
Interface PAE (4 Å)	0.93	0.90	0.81
Interface PAE (5 Å)	0.92	0.89	0.81
Interface PAE (6 Å)	0.91	0.89	0.80
Interface PAE (7 Å)	0.91	0.88	0.80
Interface PAE (10 Å)	0.91	0.88	0.80
Interface pLDDT (4 Å)	0.97	0.90	0.84
Interface pLDDT (5 Å)	0.95	0.88	0.82
Interface pLDDT (6 Å)	0.94	0.85	0.80
Interface pLDDT (7 Å)	0.93	0.83	0.77
Interface pLDDT (10 Å)	0.91	0.81	0.77

[a] Scoring methods. "Interface PAE": the average PAE score of pairs of interface residues within the interface distance cutoff specified in parenthesis; "Interface pLDDT": the average pLDDT of interface residues within the interface distance cutoff specified in parenthesis.

Table S4. Additional antibody-antigen complex test cases and AlphaFold prediction success.

PDB	Antibody	Antigen	Type[a]	AlphaFold T1[b]	AlphaFold T5[b]
5F72	LS146	KEAP1	ab	Incorrect	Incorrect
1DZB	1F9	HEW	ab	Incorrect	Incorrect
3UZE	4E11	Dengue E DIII	ab	Incorrect	Incorrect
6EJM	scFv 5	CD81 LEL	ab	Incorrect	Incorrect
5DFW	K13	CD81 LEL	ab	Incorrect	Incorrect
6I07	MM131	EpCAM	ab	Incorrect	Incorrect
5JYM	TSP11	P-cadherin	ab	Incorrect	Incorrect
4NIK	F5	Gankyrin	ab	**Medium**	**Medium**
5JYL	TSP7	P-cadherin	ab	Incorrect	Incorrect
6TOU	RVC20	Rabies gp	ab	Incorrect	Incorrect
6EK2	scFv 10	CD81 LEL	ab	Incorrect	Incorrect
4YJZ	H2526	H1 HA	ab	Incorrect	Incorrect
3UX9	AIFNα1bScFv01	interferon alpha	ab	Incorrect	Incorrect
6OAN	Antibody 053054	P vivax DBP	ab	**Medium**	**Medium**
6J71	HUA21	HER2	ab	Incorrect	Incorrect
7DET	PR961	SARS-CoV-2 RBD	ab	Incorrect	Incorrect
7DEO	PR1077	SARS-CoV-2 RBD	ab	Incorrect	Incorrect
6WAQ	VHH-72	SARS-CoV RBD	nano	Incorrect	Incorrect
6EY0	NB01	PorM	nano	Incorrect	Incorrect
6OQ7	E3	Clostridium difficile toxin B	nano	Incorrect	Incorrect

[a] Antibody type: "ab": heavy-light chain antibody, "nano": nanobody/VHH.
[b] AlphaFold modeling accuracy in top 1 and top 5 models (ranked by pTM). Model quality was assessed by CAPRI criteria. Medium CAPRI accuracy levels are highlighted with bold font.

Table S5. AlphaFold modeling success for VLR-antigen and repebody-antigen complexes.

PDB	Receptor	Antigen	Release Date[a]	AlphaFold T1[b]	AlphaFold T5[b]
3G3A	VLRB.2D	HEL	6/23/09	Incorrect	Incorrect
3M18	VLRA.R2.1	HEL	6/30/10	Incorrect	Incorrect
6BXC	VLR9	Zebrafish TLR5	5/9/18	Incorrect	Incorrect
6BXA	VLR2	Zebrafish TLR5	5/9/18	**High**	**High**
5B4P	r-3E8 repebody	Human C5a	4/12/17	Incorrect	Incorrect
6HTF	rF10 repebody	Human Btk SH2	5/20/20	Incorrect	Incorrect
6LBX	Rb-H2 repebody	HER2 Domain IV	11/18/20	Incorrect	Incorrect

[a]Release date of the experimentally determined complex structure in the Protein Data Bank.
[b]AlphaFold modeling accuracy in top 1 (T1) and top 5 (T5) models (ranked by pTM). Model quality was assessed by CAPRI criteria. High CAPRI accuracy levels are highlighted with bold font.

Table S6. AlphaFold-Multimer modeling success for recently released antibody-antigen complex structures.

PDB	Antibody	Antigen	Release Date[a]	T1[b]	T5[b]
6A4K	32D6 Fab	H1N1 hemagglutinin	3/27/19	Incorrect	Incorrect
6HX4	1D9 Fab	Alpha-1-antitrypsin	10/30/19	**Medium**	**Medium**
6P50	4A10 Fab	Interleukin-7 receptor subunit alpha	9/4/19	Incorrect	Incorrect
6PXH	G2 Fab	MERS-CoV spike NTD	9/25/19	Incorrect	Incorrect
6Q0O	H2227 Fab	H1N1 hemagglutinin	12/18/19	Acceptable	Acceptable
6U54	ZC nanobody	Ebolavirus nucleoprotein	11/6/19	**Medium**	**Medium**
6ZTR	CQY684 Fab	Cadherin-3	5/5/21	Incorrect	Incorrect

[a]Release date of the experimentally determined complex structure in the Protein Data Bank.
[b]AlphaFold-Multimer modeling accuracy in top 1 (T1) and top 5 (T5) models (ranked by AlphaFold-Multimer score). AlphaFold-Multimer was run with MSA input and without the use of structural templates. Model quality was assessed by CAPRI criteria. Medium CAPRI accuracy levels are highlighted with bold font.

Table S7. Expanded set of recent antibody-antigen complex structures used for benchmarking AlphaFold-Multimer.

PDB[a]	Antibody	Antigen	Nanobody[b]	Release Date[c]
6a0z	Fab 13D4	Hemagglutinin,Envelope glycoprotein	0	2018-06-20
6dfj	anti-Zika antibody Z021	Dengue 1 Envelope DIII domain	0	2018-10-24
6ddv	Anti-MICA Fab fragment clone 6E1	MHC class I chain-related protein A	0	2018-10-24
6mej	antibody HEPC3	E2 glycoprotein	0	2018-11-21
6e63	TB31F Fab	Pf48/45	0	2018-11-28
6h70	Nano-62	Capsid protein VP1	1	2018-12-19
6iuv	3C11	Hemagglutinin	0	2019-01-16
6a77	Anti-human Robo1 antibody B5209B Fab	Roundabout homolog 1	0	2019-01-30
6iek	Fab R12	NSmGnGc	0	2019-04-10
6dkj	hGIPR-Ab Fab	Gastric inhibitory polypeptide receptor	0	2019-05-08
6gku	Fab 6F5	Galectin-10	0	2019-06-05
6iap	Fab NKp46-1	Natural cytotoxicity triggering receptor 1	0	2019-06-12
6hhu	nanobody AF684	S-layer protein sap	1	2019-07-24
6nmv	Fab 218 anti-SIRP-alpha antibody	Tyrosine-protein phosphatase non-receptor type substrate 1	0	2019-08-07
6oy4	7A1	Der p 2 variant 3	0	2019-08-28
6hhc	FXIA ANTIBODY Fab	Coagulation factor XI	0	2019-09-11
6mg7	CH54 Fab	clade A/E 93TH057 HIV-1 gp120 core	0	2019-09-25
6phc	2544 Fab	25 kDa ookinete surface antigen	0	2019-10-02
6hjx	nanobody 72	Cys-loop ligand-gated ion channel	1	2019-10-09
6j15	GY-5 Fab	Programmed cell death protein 1	0	2019-11-06
6ir2	mCherry's nanobody LaM2	MCherry fluorescent protein	1	2019-11-13
6ir1	mCherry's nanobody LaM4	MCherry fluorescent protein	1	2019-11-13
6ppg	Fab MCAF5352A	Interleukin-17F	0	2019-12-11
6k0y	mAb059c	Programmed cell death protein 1	0	2019-12-11
6ion	anti-C4.4A antibody 11H10	Ly6/PLAUR domain-containing protein 3	0	2020-01-15
6o9i	Fab 2	Gastric inhibitory polypeptide receptor	0	2020-01-22
6vel	66E8 Fab	Ubiquitin-like protein SMT3,Cadherin-1	0	2020-01-29
6ssi	NANOBODY 22	Cys-loop ligand-gated ion channel	1	2020-02-12
6os2	Nanobody Nb.AT110i1_le	Type-1 angiotensin II receptor,Soluble cytochrome b562 BRIL fusion protein	1	2020-02-19
6uft	JLK-G12	Botulinum neurotoxin type B	1	2020-03-04
6ui1	ciA-B5	BoNT/A	1	2020-03-04
6lz9	t8E4 Fab	Hepatocyte growth factor	0	2020-03-11
6tej	Syb_NL5	Drug ABC transporter ATP-binding protein	1	2020-04-01
6obo	VHH antibody V6A6	Ricin	1	2020-04-01
6ute	Z032 Fab	Envelope domain III	0	2020-04-15
6xw7	Nanobody NB-5829	Capsid protein	1	2020-04-22
6lr7	Nanobody LaG16	Green fluorescient protein uv	1	2020-04-29
6u9s	5A6 Fab	CD81 antigen	0	2020-05-13
6p3r	Fab H5.31	Hemagglutinin	0	2020-05-27
7bwj	P2B-2F6	Spike protein S1	0	2020-06-03
6m3b	c25k23 Fab	Vitamin K-dependent protein C	0	2020-07-08
7jmp	COVA2-39	Spike protein S1	0	2020-08-26
6xkr	Sasanlimab Fab	Programmed cell death protein 1	0	2020-09-09
6wh9	1D10	KR1	0	2020-09-09
6w7s	2G10 Fab	EryAI	0	2020-09-09
7chf	BD-368-2 Fab	Spike protein S1	0	2020-09-16
6xzw	Fab 4B3	Lipoprot_C domain-containing protein	0	2020-10-14

7kfv	C1A-B3 Fab	Spike glycoprotein	0	2020-12-02
7dm2	Fab p4-170	Phosphate-binding protein PstS 1	0	2020-12-23
6ye3	UFKA-20	Interleukin-2	0	2020-12-30
7chz	IgG26A	Interleukin-1 beta	0	2021-01-13
7kn6	VHH V	Spike protein S1	1	2021-01-20
7kn5	VHH E	Spike protein S1	1	2021-01-20
7kgj	Sb45	Spike glycoprotein	1	2021-02-03
7kzb	CR3014-C8 antibody	Spike glycoprotein	0	2021-02-03
7bei	COVOX-150	Spike glycoprotein	0	2021-03-03
7lbg	Fab 13H11	Envelope glycoprotein H	0	2021-03-10
7jtg	RM11-43 Fab	Envelope glycoprotein E2	0	2021-03-10
6was	GN1_PA8 Fab	1FD6 16055 V1V2 scaffold	0	2021-03-31
7lm8	COVA1-16 Fab	Spike protein S1	0	2021-03-31
7lsg	T025 Fab	Core protein	0	2021-04-07
7c88	JS003	Programmed cell death 1 ligand 1	0	2021-04-14
7dr4	anti-human IL-2 antibody, mouse Ig G,	Interleukin-2	0	2021-04-14
6ztr	CQY684 Fab	Cadherin-3	0	2021-05-05
7m7w	Monoclonal antibody S2X259 Fab	Spike protein S1	0	2021-05-05
7cho	antibody P5A-1D2	Spike protein S1	0	2021-05-19
7mfu	Synthetic Nanobody #14 (Sb14)	Spike protein S1	1	2021-06-02
7djz	MW01	Spike protein S1	0	2021-06-09
7mhy	3H02 Fab	Protein-cysteine N-palmitoyltransferase HHAT	0	2021-06-16
7lue	ADI-14442 Fab	Fusion glycoprotein F0	0	2021-06-16
7o9s	Fab nnHTN-Gn2	Envelope polyprotein	0	2021-06-23
6ze1	G11a nanobody	Cystic fibrosis transmembrane conductance regulator	1	2021-06-30
7rnn	Nb.C1	Acid-sensing ion channel 1	1	2021-08-11
7apj	NB41	RAC-alpha serine/threonine-protein kinase	1	2021-08-25
7kyo	F100S PsaBC-Fab	Manganese ABC transporter, ATP-binding protein	0	2021-08-25
7o06	Camelid nanobody 10Z	Centrosomal protein of 164 kDa	1	2021-09-08
7e5o	NT-193	Spike protein S1	0	2021-09-08
7rah	M2B10 Fab	Bifunctional adenylate cyclase toxin/hemolysin CyaA	0	2021-09-15
7kn3	S-B8 Fab	Spike protein S1	0	2021-09-22
7mzm	PDI 215	Spike protein S1	0	2021-10-06
7mzj	PDI 93	Spike protein S1	0	2021-10-06
7anq	VHH P1.40 minibody anti-Cter PCSK9	Proprotein convertase subtilisin/kexin type	1	2021-10-20
7daa	anti-basigin Fab	Isoform 2 of Basigin	0	2021-10-20
7e72	Fab 3H7	Angiopoietin-1 receptor	0	2021-11-10
7bnv	ION-300 Fab	Surface glycoprotein	0	2021-11-17
7seg	anti-CD16A Fab	Low affinity immunoglobulin gamma Fc region receptor III-A	0	2021-11-24
7dk2	MW07	Spike protein S1	0	2021-12-08
7ps2_1	Beta-53	Spike protein S1	0	2021-12-15
7ps2_2	Beta-29 Fab	Spike protein S1	0	2021-12-15
7ps6	Beta-54 Fab	Spike protein S1	0	2021-12-15
7ps0	Beta-24	Spike protein S1	0	2021-12-15
7ps1	Beta-27 Fab heavy chain	Spike protein S1	0	2021-12-15
7lzp_1	JPU-G11	Botulinum neurotoxin A	1	2021-12-22
7q0i	Beta-43	Spike protein S1	0	2021-12-22
7na9	JSG-C1	Botulinum neurotoxin type B	1	2021-12-22
7q0g	Beta-49 Fab	Spike protein S1	0	2021-12-22

7lzp_2	JPU-B9	Botulinum neurotoxin A	1	2021-12-22
7l6v	JPU-C1	BoNT/A	1	2021-12-22
7bbj	mAb19	5'-nucleotidase	0	2021-12-29
7t5f	JLJ-G3	Botulinum neurotoxin type B	1	2021-12-29

[a]Protein Data Bank (PDB) code of the antibody-antigen complex structure. For complexes with two distinct antibody-antigen interfaces in the same PDB structure (two antibodies in complex with the same antigen), the names are differentiated with "_1" or "_2" after the PDB code.
[b]Antibody is a nanobody (1) or heavy-light chain antibody (0).
[c]Release date of the structure in the PDB.

Table S8. AlphaFold-Multimer modeling performance for T cell receptor-peptide-MHC complexes.

PDB	Release date[a]	TCR name	T1[b]	T5[b]
6l9l	11/18/2020	1D4	Incorrect	Acceptable
6mtm	2/13/2019	EM2	**Medium**	**Medium**
6r2l	2/26/2020	NYBR1	Incorrect	Incorrect
6rpa	1/15/2020	NYE_S2	Incorrect	Incorrect
6rpb	1/15/2020	NYE_S1	Incorrect	Incorrect
6rsy	4/15/2020	a7b2	Incorrect	Incorrect
6tro	6/24/2020	GVY01	Acceptable	Acceptable
6uk4	8/19/2020	302 TIL	Acceptable	Acceptable
6uln	5/27/2020	9d	Acceptable	Acceptable
6vrm	6/17/2020	12-6	Incorrect	Incorrect
6vrn	6/17/2020	38-10	Incorrect	Incorrect
7n1e	7/28/2021	pRLQ3	Incorrect	Acceptable
7n1f	7/28/2021	pYLQ7	**Medium**	**Medium**
7rm4	2/9/2022	6-11	Incorrect	Incorrect

[a]Release date of the structure in the PDB.
[b]AlphaFold-Multimer modeling accuracy in top 1 (T1) and top 5 (T5) models (ranked by AlphaFold-Multimer score). Model quality was assessed by CAPRI criteria. AlphaFold-Multimer was run with MSA input and with the use of structural templates for individual component chains, excluding structures released after 4/30/2018 as chain templates. Medium CAPRI accuracy levels are highlighted with bold font.

REFERENCES

1. Rose PW, Beran B, Bi C, et al. The RCSB Protein Data Bank: redesigned web site and web services. *Nucleic acids research.* 2011;39(Database issue):D392-401.
2. Leman JK, Weitzner BD, Lewis SM, et al. Macromolecular modeling and design in Rosetta: recent methods and frameworks. *Nat Methods.* 2020;17(7):665-680.
3. Pierce B, Weng Z. A combination of rescoring and refinement significantly improves protein docking performance. *Proteins.* 2008;72(1):270-279.
4. *NACCESS* [computer program]. Version 2.1.1: Department of Biochemistry and Molecular Biology, University College London; 1993.
